# PIP5K isoforms and phosphoinositide molecular species in EGF-stimulated PIP_3_ synthesis: A multi-method approach using enzyme genetic suppression and chemical inhibition in MCF10A cells

**DOI:** 10.1371/journal.pone.0362260

**Published:** 2026-11-05

**Authors:** Iosif Alexandrou, Vikki Flemington, Martin E. Swarbrick, Sabina Cosulich, Phillip T. Hawkins, Len R. Stephens

**Affiliations:** 1 Babraham Institute, Cambridge, United Kingdom; 2 Astra Zeneca, Cambridge, United Kingdom; 3 Cancer Research Horizons Therapeutic Innovation, Babraham Research Campus, Cambridge, United Kingdom; National Cancer Institute, UNITED STATES OF AMERICA

## Abstract

The membrane phospholipid PI(4,5)P_2_ has structural functions and is a substrate for PLC and PI3K enzymes to generate Ins(1,4,5)P_3_, DAG and PI(3,4,5)P_3_ signals. PIP5Ks, that 5-phosphorylate PI4P, are the major source of PI(4,5)P_2_ in mammalian cells. Current thinking suggests distinct pools of PI(4,5)P_2_ may be differentially generated by PIP5Kα, β and γ isoforms and involved in different physiological functions. Phosphoinositides are comprised of families of acyl-chain variants but are uniquely enriched in C38:4 species. There is evidence that PIP5Ks are selective for certain acyl chain species of PI(4)P. We aimed to understand how inhibition of PIP5Ks impacts PIP_3_ production and whether they differentially support synthesis of a pool of PI(4,5)P_2_, that may be marked by a distinct profile of molecular species variants, which supply class I PI3Ks in a non-transformed human cell line (MCF10A cells). Using a combination of siRNA, Cas9/CRISPR-mediated gene knockout (k/o), recently derived selective, small-molecule inhibitors of PIP5Ks and mass-spectrometry-based lipidomic techniques, we attempted to address these questions. We found that MCF10A cells only express PIP5Kα and γ and that neither siRNA-mediated reduction nor k/o of either protein changed the levels or acyl chain profiles of any of the phosphoinositides in Epidermal Growth Factor- (EGF) stimulated MCF10A cells. We found that selective PIP5K inhibition reduced levels of PIP_2_ and EGF-stimulated PIP_3_ and increased levels of PIP, however, the acyl-chain-profiles of none of these lipids were significantly altered in MCF10A cells. We found EGF-stimulated Akt, EGF Receptor (EGFR) and MAPK phosphorylation were also reduced by PIP5K inhibitors in this cell line. To deconvolute these results, we tested the impact of a selective PI3Kα inhibitor (A66) and found it inhibited PIP_3_ accumulation and Akt phosphorylation and reduced MAPK phosphorylation but was without effect on total EGFR or its phosphorylation, suggesting some cross-talk between EGF-stimulated activation of class I PI3K and MAPKs in MCF10A cells.

## Introduction

Phosphatidylinositol (3,4,5)-trisphosphate (PtdIns(3,4,5)P_3_, PI(3,4,5)P_3_ or PIP_3_) is an intracellular signalling lipid that controls a huge range of metabolic and functional responses. In broad terms, for many cell and tissue types, these cellular responses are anabolic [1]. PIP_3_ is synthesised by class I PI3Ks that 3-phosphorylate the polyphosphoinositide, PI(4,5)P_2_. There are 4 class I PI3Ks in mammals, α, β, δ, and γ. They are all heterodimers comprised of regulatory and catalytic subunits (α, β, δ, and γ that give their name to host class I PI3K complex). They can be activated by cell surface receptors, including EGF receptor, as a result, PIP_3_ levels rise dramatically. PIP_3_ is removed from cells by PIP_3_-phosphomonoesterases, significantly including the PIP_3_–3-phosphatase, PTEN. Unsurprisingly, the majority of human tumours derive benefit from mutations that increase the activity of this network, most commonly in activating mutations in the class I PI3Ks or through loss of PTEN activity. As a result, the class I PI3K/PIP_3_ signalling network has been heavily targeted to discover potential anti-cancer drugs [2].

PI(4,5)P_2_ is also the substrate for phospholipases C (PLCs or phosphoinositidases C (PICs)), that can be activated by a variety of receptors, to generate the intracellular messengers Ins(1,4,5)P_3_, which mobilises intracellular Ca^2+^, and DAG (diacylglycerol), which activates some members of the PKC-family of protein kinases [3]. PI(4,5)P_2_ also binds and/or regulates the activity of a wide range of signalling and scaffold proteins. As a result of these diverse critical cellular roles the levels of PI(4,5)P_2_ are controlled tightly. PI(4,5)P_2_ is localized largely to the plasma membrane and is often considered a marker and key determinant of the plasma membrane compartment [4]. There is, however, strong evidence showing PI(4,5)P_2_ can be detected in a number of other cellular membranes [5].

PI(4,5)P_2_ is synthesised predominantly by PI4P 5-kinases (PIP5Ks). There are 3 versions of PIP5K in mammals; PIP5Ks α, β, and γ, that are subject to a variety of forms of regulation including, small GTPases [6]. In turn, PI4P can be generated by 4-phosphorylation of PtdIns (or by removal of the 5-phosphate from PI(4,5)P_2_). A family of PIP4Ks synthesise distinct but minor pools of PI(4,5)P_2_ in specific intracellular locations including the nucleus [6,7].

PI(4,5)P_2_ is also degraded by 5-phosphate-directed phosphomonoesterases [8]. The outcome of these activities is that PI(4,5)P_2_ can be turned over relatively rapidly and this underlies the fact that the monoester phosphate groups of PI(4,5)P_2_ (and PI(4)P) are turned-over rapidly and label very quickly with [^32^P]-Pi [9].

In the face of intense activation of PLCs, the rate of consumption of PI(4,5)P_2_ could lead to its total depletion within minutes [9–12]. However, PI(4,5)P_2_ levels are only reduced transiently before returning towards control levels despite continued PLC-activation as a result of a coordinated increase in its net synthesis. It is likely that PIP5K activity is enhanced during this period of high consumption by a combination of factors including released product inhibition [13,14] and receptor-activation possibly *via* small GTPases [15,16]. It is also likely that PI(4,5)P_2_-phosphomonoesterase activity is reduced by substrate depletion. Finally, depletion of PI(4,5)P_2_ levels can also work to enhance the availability of PI4P in the plasma membrane for its re-synthesis (through the ability of PI(4,5)P_2_ levels to control Orp5/8 recruitment – a PI4P / PS counter transporter that takes PI4P from the plasma membrane to the ER and returns PS – to the plasma membrane in areas of close proximity with the ER) [17]. In combination these factors can support a large increase in PI(4,5)P_2_ synthesis upon its PLC-induced consumption. There is little evidence that PI(4,5)P_2_ levels can be significantly depleted by activation of PI3Ks alone. PIP5K-isoform selective small molecule inhibitors have been described recently that can inhibit PI(4,5)P_2_ synthesis in cells [18,19].

Phosphoinositides, like other cellular phospholipids, are comprised of families of molecular species variants, that contain the same relevant head group but distinct fatty acids. A unique feature of the phosphoinositides, including PIP_3_, is that their profile of fatty acids is particularly homogenous with substantial enrichment in C38:4-species (where characterised this is very largely made up of stearoyl (SN1) and arachidonoyl (SN2) fatty acids) [20]. Some enzymes, involved in phosphoinositide metabolism have been reported to be more selective for substrates with stearoyl-arachidonoyl moieties, such as DAG kinase ε, which converts DAG to phosphatidic acid (PA), and CDP-diacylglycerol synthetases, the enzymes that convert PA into CDP-DAG [21,22]. PIP5Kα was found to be able to phosphorylate stearoyl-arachidonoyl-PI(4)P much faster than dipalmitoyl-PI(4)P *in vitro*, and it was proposed that a specific amino-acid motif in PIP5Ks, which exists also in DAG kinase ε, may confer selectivity towards arachidonate-containing species. Further work showed all the PIP5K isoforms showed preference for species with one unsaturated lipid moiety, such as stearoyl-arachidonoyl and stearoyl-oleoyl (C18:0/C18:1) substrates over saturated species [23,24]. However, these examples of apparent intrinsic acyl chain selectivity by enzymes, including PIP5Ks, could be a result of the different molecular species of their substrates, e.g., PI(4)P, being subtly differently distributed within the assay environment, i.e., they may be differentially available as substrates rather than being discriminated directly by the PIP5Ks. The factors leading to their differential availability *in vitro* may not be relevant *in vivo* and hence whether PIP5Ks preferentially utilise stearoyl-arachidonoyl-PI(4)P species *in vivo* is unclear.

Recent work has shown that there can be small and/or differential changes in the acyl composition of phosphoinositides, particularly PIP_3_, upon stimulation [25,26], indicating there may be some selection by either PI3Ks or PIP_3_-phosphatases *in vivo*. However, a recent study of the metabolism of phosphoinositides using isotopologue tracing in intact cells suggested that the C38:4 signature was a result of a combination of C38:4-selective reactions in acyl chain remodelling, via LPAIT1 and LYCAT, and PI-cycle mediated salvage of PLC-generated DGs, at the levels of DG kinases and CDS enzymes, but found no evidence of acyl-chain selective phosphorylation of PI to PIP_2_ [27]. Hence, whether PIP and PIP_2_ (PI3Ks) kinases show acyl chain selective use of their substrates *in vivo* remains unclear.

A significant body of evidence indicates the different PIP5K variants can have unique roles in generating PIP_2_ pools that support PLC and PI3K signalling pathways, however, the exact PIP5K required also seems to depend on the cell context. During keratinocyte differentiation, the cell adhesion complex molecules recruit and activate PIP5Kα at the cell membrane. PIP_2_, produced by that isoform, appears to be preferentially used by PI3Ks as the substrate for PIP_3_ production, as well as by PLC-γ1 as the substrate for DAG and IP_3_ generation [28]. A PIP5Kγ splice variant was reported to be the major PIP5K which supplies the PLC pathway with PIP_2_ in HeLa cells upon stimulation by histamine, whereas the PIP5Ks α and β do not seem to play an important role [29]. However, in mouse platelets, activated by thrombin, PIP5Ks α and β, but not γ, contribute to the PLC-sensitive PIP_2_ pool [30]. A recent study reported that in human breast cancer cells, the splice variant PIP5Kγ-i2 and PI3K can assemble as parts of a complex. In this way PIP5Kγ-i2 provides a local PIP_2_ pool, used by PI3K for PIP_3_ production and oncogenic downstream effects [31]. Finally, a specific complex involving IQGAP, PIP5Kα and class I PI3Ks has been described that supports stimulated accumulation of PIP_3_ and activation of AKT [32].

It has been suggested that the multiple functions of PI(4,5)P_2_ indicate the presence of different PI(4,5)P_2_ pools in the cell, which are associated with specific functions and downstream effectors. Some of these pools may be generated by different PIP5K isoforms, with distinct sub-cellular localizations. It is possible that these functionally different PI(4,5)P_2_ pools may be differentially enriched in different PI(4,5)P_2_ molecular species. The current work intended to provide insights into the role of specific PIP5K isoforms and phosphoinositide molecular species in the class I PI3K pathway, using normal human breast epithelium MCF10A cells (called wild-type MCF10A cells in this work). We also reasoned it would be important to understand whether the same or a related potential pool of PI(4,5)P_2_ would also be used by PLCs.

## Materials and methods

### Cell cultures

Immortalized, normal human breast epithelium MCF10A cells (ATCC, CRL-10317) were maintained (37°C, 5% CO_2_), in DMEM/F12 (Life Technologies), supplied with 5% horse serum (PAA), penicillin and streptomycin (94 units/ml, Life Technologies), epidermal growth factor (EGF) 10 ng/ml (Sigma-Aldrich), insulin (10 μg/ml, Sigma-Aldrich), cholera toxin (0.1 μg/ml, Sigma-Aldrich), and hydrocortisone (0.5 μg/ml, Sigma-Aldrich). Cells were starved by washing with PBS and overnight incubation (16–18 hours, 37°C, 5% CO_2_) in DMEM/F12, supplied with 1% charcoal-stripped foetal bovine serum (PAA), cholera toxin 0.1 μg/ml, and hydrocortisone 0.5 μg/ml.

### Stearoyl-arachidonoyl-DAG measurement

After overnight starvation, MCF10A cells were stimulated with human EGF (10 ng/ml) or the EGF vehicle (mock stimulation) for 15 or 30 seconds, 1 or 2 minutes at 37°C, 5% CO_2_ before quenching with ice-cold methanol-water 1:1. DAG was extracted at room temperature by adding 122 μl methanol, 974 μl chloroform and 10 μl of deuterated stearoyl-arachidonoyl-DAG as internal standard (1 pg/μl in isopropyl-alcohol, Babraham Institute). After mixing and centrifugation (6500 rpm, 5 minutes), 1 ml of the lower organic phase was transferred into 12 × 32 mm glass screw neck vials (Waters). The samples were dried under nitrogen (20 μl leftover volume) before adding 100 μl methanol-water 4:1. Stearoyl-arachidonoyl-DAG was measured by mass spectrometry as previously described [33].

### Ca^2+^ imaging assays

MCF10A cells were starved overnight on sterile 22 mm glass coverslips (VWR) before incubation with 1 μM Fura-2 Ca^2+^ indicator (Life Technologies) in imaging buffer (sodium chloride 121 mM, potassium chloride 5.4 mM, magnesium chloride 0.8 mM, calcium chloride 1.8 mM, sodium bicarbonate 6 mM, glucose 5.5 mM, HEPES 25 mM, pH 7.5) for 30 minutes at room temperature. The cells were washed and incubated in imaging buffer for 30 minutes before the imaging started. During imaging, buffer was added at 30 seconds (negative control) before stimulation with EGF (10, 40, or 100 ng/ml) at 60 seconds. At the end of the eleventh minute, 10 or 100 μM Adenosine 5′-triphosphate disodium salt hydrate (Sigma-Aldrich) was added as a positive control. Images were taken for 13 minutes totally with an Olympus IX81 wide-field epi-fluorescence microscope, equipped with a monochromator light source and monochrome camera. Signal was quantified (ImageJ) and the average Ca^2+^ response from ten regions of interest was plotted over time as 340/380 nm signal ratio after subtracting background signal.

### IP3 measurement

MCF10A cells were washed twice and starved for 5 hours in inositol-free DMEM/F12 medium (BioConcept), supplied with L-glutamine 2.5 mM, dialysed bovine serum 1% (Sigma-Aldrich), cholera toxin 0.1 μg/ml, and hydrocortisone 0.5 μg/ml. The cells were then starved in the inositol-free medium for 5 hours. After washing with the same type of medium, they were starved overnight in inositol-free medium, supplied with 200 μCi myo-[2 3H(N)]-inositol, (21.7 Ci/mmol, Perkin-Elmer) and 16 mM sodium chloride. Finally, the cells were washed with and pre-incubated with 20 mM LiCl for 30 minutes in non-radiolabelled inositol-free medium to inhibit inositol 1-phosphate phosphatase activity before stimulation with EGF (10 ng/ml) or its vehicle (mock stimulation). Stimulation was quenched in 800 μl ice-cold perchloric acid 5% (Sigma-Aldrich). After mixing and centrifugation (12,000 rpm, 10 minutes, 4°C), 750 μl of supernatant were neutralized with 690 μl 1:1 FREON/tri-N-octylamine mixture (VWR, Acros Organics). The samples were supplemented with EDTA (10 μl of 100 mM solution, pH 7.55), mixed and centrifuged (5,000 rpm, 4 minutes, room temperature). Approximately 670–700 μl of upper aqueous phase were diluted in ultrapure water up to 3 ml. Inositol phosphates were separated by anion-exchange column chromatography through Dowex resin (1 × 8, 200–400 mesh, Sigma-Aldrich), and the radioactivity was measured as decays per minute (dpm) in a Packard liquid scintillation analyser (model TRICARB 1600).

### PIP5Kα and PIP5Kγ suppression by siRNAs

Pools of four PIP5Kα-targeting (ON-TARGET*plus* SMARTpool, Dharmacon, L-004780-00-0020) or PIP5Kγ-targeting siRNAs (Dharmacon, L-004782-00-0020) and a non-targeting siRNA pool (Dharmacon, D-001810-10-20) were used for transfection. Briefly, siRNA pools were pre-incubated for 15 minutes at room temperature with appropriate volume of transfection reagent DharmaFECT 1 (Dharmacon, T-2001–03) in Opti-MEM reduced-serum medium (Life Technologies). The mixture was added in MCF10A cell suspension in Opti-MEM, making a final siRNA concentration of 78 nM, before plating in 35 mm dishes (200,000 cells per dish). Opti-MEM was replaced by antibiotics-free normal culture medium after 8 hours, and the cells were incubated for 48 hours (37°C, 5% CO_2_).

### Lipid extraction and mass spectrometry lipid measurement

After overnight starvation and stimulation with 10 ng/ml EGF or its vehicle (mock stimulation), the samples were quenched in 500 μl ice-cold hydrochloric acid 1 M. One third of each cell lysate sample was centrifuged (12,000 rpm, 4°C, 5 minutes) and processed for lipid extraction, methylation using trimethylsilyl-diazomethane, and mass spectrometry measurement following a previously described protocol [26]. Synthetic PIP3 (1 ng) and PI (100 ng), produced at the Babraham Institute, were spiked in the samples as internal standards (ISD). PIP_3_ and PIP_2_ signals were normalised over ISD PIP_3_ signal, whereas PIP and PI signals were normalised over ISD PI.

### Chemical inhibition assays

PIP5K inhibitors were provided by AstraZeneca/Cancer Research Horizons and prepared as 10 mM stock solutions in dimethyl-sulfoxide (DMSO, Sigma Aldrich, D2650), stored at room temperature and protected from light. A66 PI3K inhibitor was purchased from Selleckchem. MCF10A cells (200,000 cells per 35 mm dish) were incubated for 24 hours in normal growth medium and starved overnight. After washing with PBS, they were incubated with various compound concentrations or its vehicle (1% DMSO) in starvation medium (DMEM/F12, supplied with 1% charcoal-stripped foetal bovine serum (PAA), cholera toxin 0.1 μg/ml, and hydrocortisone 0.5 μg/ml). Finally, the cells were stimulated with human EGF (10 ng/ml in starvation medium) or its vehicle (mock stimulation). The stimulation was quenched in 500 μl ice-cold hydrochloric acid 1 M. One third of each cell lysate volume was processed for lipid extraction and mass spectrometry measurement, as previously described.

### Generation of PIP5Kα and PIP5Kγ k/o cell lines

Target sites were selected using the online tool http://tools.genome-engineering.org [34]. Designed oligonucleotides (Sigma Aldrich) with a 20-nucleotide guide sequence and a 5′ overhang were ligated into the BbsI restriction site of the pSpCas9(BB)-2A-GFP (PX458) plasmid (gift from Feng Zhang, Addgene plasmid # 48138, https://www.addgene.org/48138/). Exon 1 of *PIP5Kα* and *PIP5Kγ* was targeted by the oligonucleotide 5`-3` sequences CACCGAAGGTACAGGAAGGGACCGC and CACCGGGAGCTGGAGGTACCGGACG respectively. DH5α *E coli* competent cells (Babraham Institute) were transformed with the ligation products by heat-shock at 42°C for 30 seconds. The cells were incubated for an hour in Luria-Bertani (LB) and grown overnight on LB agar plates containing 100 μg/ml ampicillin at 37°C. Single colonies were screened for the presence of the inserted oligonucleotides through PCR (HotStartTaq DNA Polymerase kit, QIAGEN), using a U6 promoter oligonucleotide as a forward primer (5′-GAGGGCCTATTTCCCATGATTCC-3′, Sigma Aldrich), and the respective reverse oligonucleotide as the reverse primer. Colonies producing a 270 base pair band on agarose gel, were grown overnight at 37°C and a cell suspension aliquot was used for DNA extraction (QIAprep Spin Miniprep Kit, QIAGEN) and sequencing (Cogenics Lark UK) to confirm the successful insertion of oligonucleotide guides. Confirmed colonies were further grown overnight and DNA was extracted using the EndoFree Plasmid Maxi Kit (QIAGEN). Wild-type MCF10A cells at an early passage (350,000 cells per well) were transfected with 2.5 μg of purified Cas9 plasmid and 1 μg of puromycin resistance plasmid (kind gift from Dr Oliver Florey, Babraham Institute), pre-incubated with Freestyle transfection reagent (Invitrogen, kind gift from Dr Dominik Spensberger, Babraham Institute) in F12 medium for 30 minutes at room temperature. After 24-hour incubation, the cells were selected by puromycin incubation (Sigma Aldrich, kind gift from Dr Dominik Spensberger) at 2.5 μg/ml for 48 hours. Puromycin-resistant cells were then grown until confluent in MCF10A-conditioned medium. The mixed cell populations were screened for PIP5K expression by immuno-blotting and those with at least 50% reduction were selected for monoclonal population generation through serial dilutions in 96-well plates. Populations developed from single cells were transferred to 6-well plates in MCF10A-conditioned medium. When confluent, they were screened for PIP5K expression by immune-blotting, and those without any detectable PIP5Kα or PIP5Kγ expression were further expanded.

### Sequencing of PIP5Kα exon 1

Cells from confluent flasks were lysed in 100 mM Tris pH 8, 5 mM EDTA, 100 mM sodium chloride, 0.2% SDS, and 100 μg/ml proteinase K (Roche, 03115801001, kindly provided by Dr Dominik Spensberger). The samples were mixed with isopropanol, centrifuged, and the pellets were washed with 70% ethanol and dried. Primers were designed using the NCBI online tool (http://www.ncbi.nlm.nih.gov/tools/primer-blast/; forward 5′-TAAACTTGTGGAGGGGGTGC-3′, reverse 5′-CCCTACCCCCACCTTCAATC-3′, Sigma Aldrich). Exon 1 was amplified using the HotStartTaq Polymerase Kit (QIAGEN, 203205) and the successful amplification was confirmed by a 567 base pair band. The PCR products were purified by the QIAquick PCR Purification Kit (QIAGEN, 28104) and cloned using the CloneJET PCR Cloning Kit (Thermo Scientific, K1231) according to manufacturer instructions. DH5α transformation and agar plate preparation were done as previously described. Colonies were picked and enhanced. The PCR products were purified and sequenced using the same primers.

### Immuno-blotting

After harvesting cells with 1 M hydrochloric acid, as previously described, cell lysate samples were centrifuged (12,000 rpm, 4°C, 10 minutes) and pellets were washed in 500 μl hydrochloric acid 10 mM, before being dissolved in 100 μl loading buffer (2% SDS, 0.1 M dithiothreitol, 40 mM Tris pH 6.7, glycerol 12.5%, bromophenol blue 0.003%). The samples were denatured at 95°C for 10 minutes and run in 1 mm SDS-PAGE stacking gel (125 mM Tris base, 0.1% SDS, 5% acrylamide, 0.1% ammonium persulfate, 0.1% Temed) and separating gel (375 mM Tris base, 0.1% SDS, 8% acrylamide, 0.1% ammonium persulfate, 0.1% Temed). Proteins were transferred (50 V, 2 hours or 35 V, overnight, 4°C) onto Immobilon-FLPVDF (polyvinylidene fluoride) membrane (Millipore, IPFL00010). Membranes were blocked in TBS (20 mM Tris base, 137 mM sodium chloride, pH 8) with 0.1% Tween20 (Sigma-Aldrich) and 5% milk (1 hour, room temperature or overnight, 4°C). Primary antibodies, used, were mouse anti-phospho-308-AKT (Cell Signalling, 5106), rabbit anti-phospho-473-AKT (Cell Signalling, 4058), goat anti-PIP5Kα (Santa Cruz Biotechnology Inc, sc-11774), goat anti-PIP5Kβ (Santa Cruz Biotechnology Inc, sc-11778), rabbit anti-PIP5Kγ (Cell Signalling, 3296), mouse anti-phospho-MAPK (Cell Signalling, 9106), rabbit anti-phospho-EGFR (Cell Signalling, 2234), mouse anti-βCOP (a kind gift to Dr Nicholas Ktistakis from the late Thomas Kreis). Secondary antibodies were goat anti-mouse (LI-COR Biosciences, 926–32210), goat anti-rabbit (LI-COR Biosciences, 926–68071), donkey anti-goat (LI-COR Biosciences, 926–32214), goat anti-mouse (Bio-Rad, 170–6516), donkey anti-goat (Santa Cruz Biotechnology Inc, sc-2020). ECL detection kit was used according to manufacturer’s instructions (GE Healthcare, RPN2232) for HRP-conjugated secondary antibodies. Signal from fluorescent secondary antibodies was recovered by LI-COR Odyssey infrared imager and quantified using the Image Studio Ver2.1 software. βCOP was probed separately as a loading control after rehydration of membranes (30 minutes in TBS + 0.1% Tween20). Cell lysate from K562 cells as a PIP5Kβ positive control was purchased from Santa Cruz Biotechnology (sc-2203).

### Statistical analysis

GraphPad Prism software was used for all statistical analysis methods, described in text or legends.

## Results

### Does EGF stimulate PLC activation in MCF10A cells?

Given our aims, described above, an initial question was whether we could identify agonists capable of stimulating activation of both PLC and class I PI3K-mediated consumption of PI(4,5)P_2_ in MCF10A cells. In the context of past evidence from our lab that EGF can activate far greater accumulation of PIP_3_ than insulin in MCF10A cells [35], we tried to establish whether EGF could stimulate PLC-activation in parallel with class I PI3K activation in MCF10A cells. To achieve that we used 3 totally independent assays; measurement of basal and EGF-stimulated cytosolic free Ca^2+^ (the most sensitive of these assays), stearoyl/arachidonoyl-DAG, as the species known to be preferentially generated by receptor-stimulated PLC activity [27,36], and [^3^H]-inositol phosphates. We found no evidence that EGF, across a large range of doses could stimulate an increase in free cytosolic Ca^2+^ (in the context that ATP could stimulate a large increase in cytosolic free Ca^2+^, S1 Fig). We found that EGF did not stimulate a significant accumulation of stearoyl-arachidonoyl-DAG (S1 Fig). Finally, EGF could not stimulate significant accumulation of [^3^H]-inositol trisphosphate ([^3^H]-InsP_3_) (S1 Fig). Further experiments with LiCl pre-incubation to inhibit degradation of inositol phosphates before a prolonged 15-minute stimulation produced IP_3_ signal close to background. From these results, and in contrast to much literature demonstrating EGF can stimulate PLC activation in cells over-expressing EGFRs, we concluded that although EGF can activate class I PI3Ks in MCF10A cells, it cannot stimulate activation of PLCs in this setting. Given the discovery that ATP could stimulate an increase in cytosolic free Ca^2+^, presumably via activation of PLC signalling, we tested whether ATP could stimulate accumulation of PIP_3_ in MCF10A cells. We found no evidence that ATP could stimulate significant accumulation of PIP_3_ (S1 Fig). Furthermore, although insulin only stimulates PI3K activation in MCF10A cells weakly, we tested whether it could cause an increase in intracellular free Ca^2+^. We found no evidence insulin could stimulate an increase in intracellular free Ca^2+^ (S1 Fig). This meant we would not be able to conduct experiments that addressed the potential differential roles of PIP5Ks in parallel, simultaneous supply of PIP_2_ to PLCs and class I PI3Ks in MCF10A cells. Hence, the remaining work focused on asking whether there was evidence of differential roles for PIP5Ks and pools of PIP_2_, potentially comprised of distinct molecular species, in supporting EGF-stimulated accumulation of PIP_3_ compared to producing PIP_2_.

### Expression of PIP5Ks in MCF10A cells

Previously published mRNA-seq data from our lab has shown that MCF10A cells express mRNAs encoding PIP5Ks α and γ but not β [37]. Immuno-blotting confirmed these data (Fig 1), with both PIP5Ks α and γ appearing as multiple bands; PIP5Kα typically as 5 bands between 45–85 kDa (databases suggest 4 splice variants from 58–62 kDa); PIP5Kγ typically as 6 bands between 80–105 kDa (databases suggest 4 splice variants from 70–77 kDa), confirmed by siRNA suppression (see below). The molecular explanation for the multiple bands for either isoform remains unclear, but it seems unlikely to be solely due to phosphorylation.

**Fig 1:**
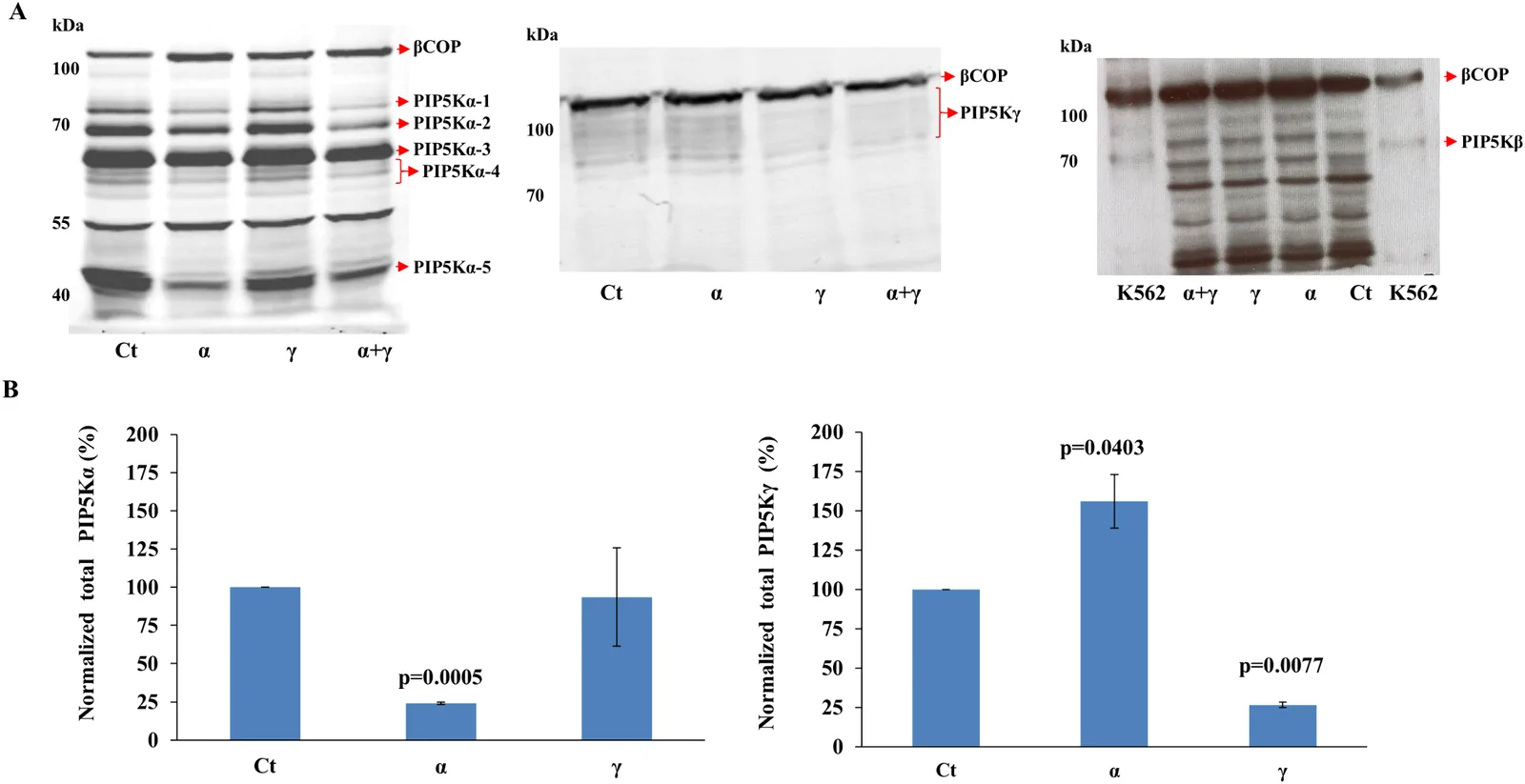
Characterization of PIP5Kα and PIP5Kγ variants in wild-type MCF10A cells with siRNAs. A: lysates from MCF10A cells, treated with non-targeting/control, PIP5Kα-, PIP5Kγ-, and PIP5Kα- and PIP5Kγ-targeting siRNA pools (Ct, α, γ, α + γ respectively) for 48 hours, were probed for PIP5Kα and βCOP as loading control (left panel). PIP5Kα variants 1-5 are identified (PIP5Kα-4 appears as a cluster of tightly packed bands). Similar samples (middle panel) were probed for PIP5Kγ (red) and βCOP (green). PIP5Kγ variants appear as a cluster of 6 tightly packed bands. PIP5Kβ remains unexpressed after any siRNA treatment (right panel). A 70 kDa band corresponding to PIP5Kβ, was detected in positive control samples (K562 cell lysate, first and last lane), whereas it was not detected in lysates, treated with PIP5Kα- (α), PIP5Kγ- (γ), PIP5Kα- and PIP5Kγ- (α + γ), or non-targeting control (Ct) siRNA pools. The major band at around 100 kDa corresponds to βCOP (loading control). B: Quantification of PIP5Kα and PIP5Kγ expression after PIP5Kα- (α), PIP5Kγ- (γ), or non-targeting/control (Ct) siRNA treatment. Fluorescent signals were normalized over βCOP (loading control) and are presented as % of the respective signal in samples treated with Ct siRNA. The graphs present means and standard errors from three independent experiments. Ratio Paired t-test compared normalised expression between control and either α or γ siRNA treatment, and significant results are identified by the p value. The first column presents control samples (Ct) as 100%.

### RNAi suppression of PIP5Ks α and γ in MCF10A cells

Following numerous experiments to optimize the efficiency of knockdown, we settled upon a single transfection with PIP5K α- or γ- or α- and γ-, or control-directed siRNA smart pools and a 48hr incubation prior to stimulation or further treatments. Under these conditions PIP5Kγ-directed siRNA pools reduced expression of a series of 6 immuno-decorated bands by an average of 73%, compared to control siRNA-treated cells, across many experiments (Fig 1). The 6 bands were assumed to represent authentic PIP5Kγ and their sum to be total PIP5Kγ. None of the variants was changed significantly differently to the total by PIP5Kγ-directed siRNA (S2 Fig). Expression of PIP5Kα (see below) was unchanged. PIP5Kβ remained undetectable.

Under the same conditions PIP5Kα-directed siRNA reduced the expression of a series of 5 protein variants identified in the PIP5Kα-directed immuno-blots, that were assumed to represent authentic PIP5Kα variants, by an average of 76% (their combined intensity was considered total PIP5Kα) compared to control siRNA-treated cells across many experiments. None of the PIP5Kα variants were changed in a significantly different fashion compared to total PIP5Kα (S2 Fig). Expression of PIP5Kγ was increased significantly by an average of 56%. PIP5Kβ remained undetectable.

Treatment with both PIP5K α- and γ-directed siRNA together (with a total siRNA concentration the same as in the experiments with the individual siRNAs) resulted in a reduction in expression of both PIP5K α and γ by an average of 73%.

### Changes in PI, PIP, PIP_2_ and PIP_3_ following siRNA suppression of PIP5K α and/or γ

Past work in our lab has shown that PIP_2_ measured by our mass spectrometry approach in parental (i.e., not PTEN-null) control or EGF-stimulated MCF10A cells is at least 95% PI(4,5)P_2_ [38] and hence although we use lipidomic techniques to measure the levels of phosphoinositides that can resolve PI, PIP, PIP_2_ and PIP_3_ and their acyl-chain molecular species, but none of their regio-isomers (e.g., PI(3,4)P_2_, PI(3,5)P_2_ and PI(4,5)P_2_), we assume that our PIP_2_ measurements represent the sum of regio-isomers and reflect mostly the levels of PI(4,5)P_2_. Similarly, our PIP recoveries represent the sum of regio-isomers (PI4P, PI3P, PI5P).

Wild-type MCF10A cells that had been treated with control, PIP5Kα or PIP5Kγ siRNA were stimulated with vehicle or EGF for various times, then quenched, and phosphoinositides were quantified by mass spectrometry. Recoveries were corrected using relevant internal standards (ISD) and normalised for input over the total amount of PI measured in the same sample (except for the PI measurements which were only corrected with the PI internal standard). This revealed that PI, PIP, PIP_2_ and PIP_3_ (the latter in EGF stimulated samples) were comprised of a number of molecular species. The precise number of molecular species variants of phosphoinositides we detected varied slightly between experiments, particularly those species that were near the limit of quantification, but here we report on a common set that could be reliably quantified in every experiment. We report “total” phosphoinositides as the sum of the amounts of the individual species. The relative quantities of the individual molecular species variants are reported as % of the relevant total phosphoinositide, measured in samples treated with control siRNA and in the absence of EGF stimulation.

Suppression of PIP5Kα (with the compensatory increase in PIP5Kγ) did not reduce total PI or PIP_2_ levels but marginally increased total PIP_3_ and PIP levels (Fig 2). These changes were similar for all the molecular species quantified with none significantly different from the changes seen in the total amount of the relevant phosphoinositide (S3A-S3D Figs). Suppression of PIP5Kγ did not clearly change the total levels of PIP_3_, PIP_2_, and PIP (Fig 3) nor any of the individual molecular species (S4A-S4C Figs). However, an unexpected increase in total PI and individual molecular species was observed in samples stimulated with EGF for 3 minutes, whereas no clear change was seen in the rest of the time points (S4D Fig). Moreover, combined suppression of PIP5Kα and γ led to a reduction in PI levels that distorted the normalisation and quantification of the polyphosphoinositides. This occurred without a significant reduction in cell viability. Normalisation of PIP_3_, PIP_2_, and PIP recoveries over PI, which was reduced because of experimental perturbation, could lead to artificial increase of measured recoveries, thus masking the true effect of combined suppression of PIP5Kα and PIP5Kγ. Moreover, without normalisation to PI, the error on the measurements of the phosphoinositides became substantial, such that it was very challenging to reliably detect a 20% change in their levels. We attempted to normalise the data with alternative estimates of cell input (e.g., protein, or use of βCOP measurements or parallel measurements of other phospholipids) but they failed to reduce the error sufficiently to afford a viable alternative. As a result, we could not satisfactorily determine whether combined suppression of PIP5Ks significantly changed the levels of any of the polyphosphoinositides. We assumed a combination of residual PIP5Kα and γ expression, isoform redundancy, and potential compensatory changes in expression or activity, led to this lack of phenotypes.

**Fig 2:**
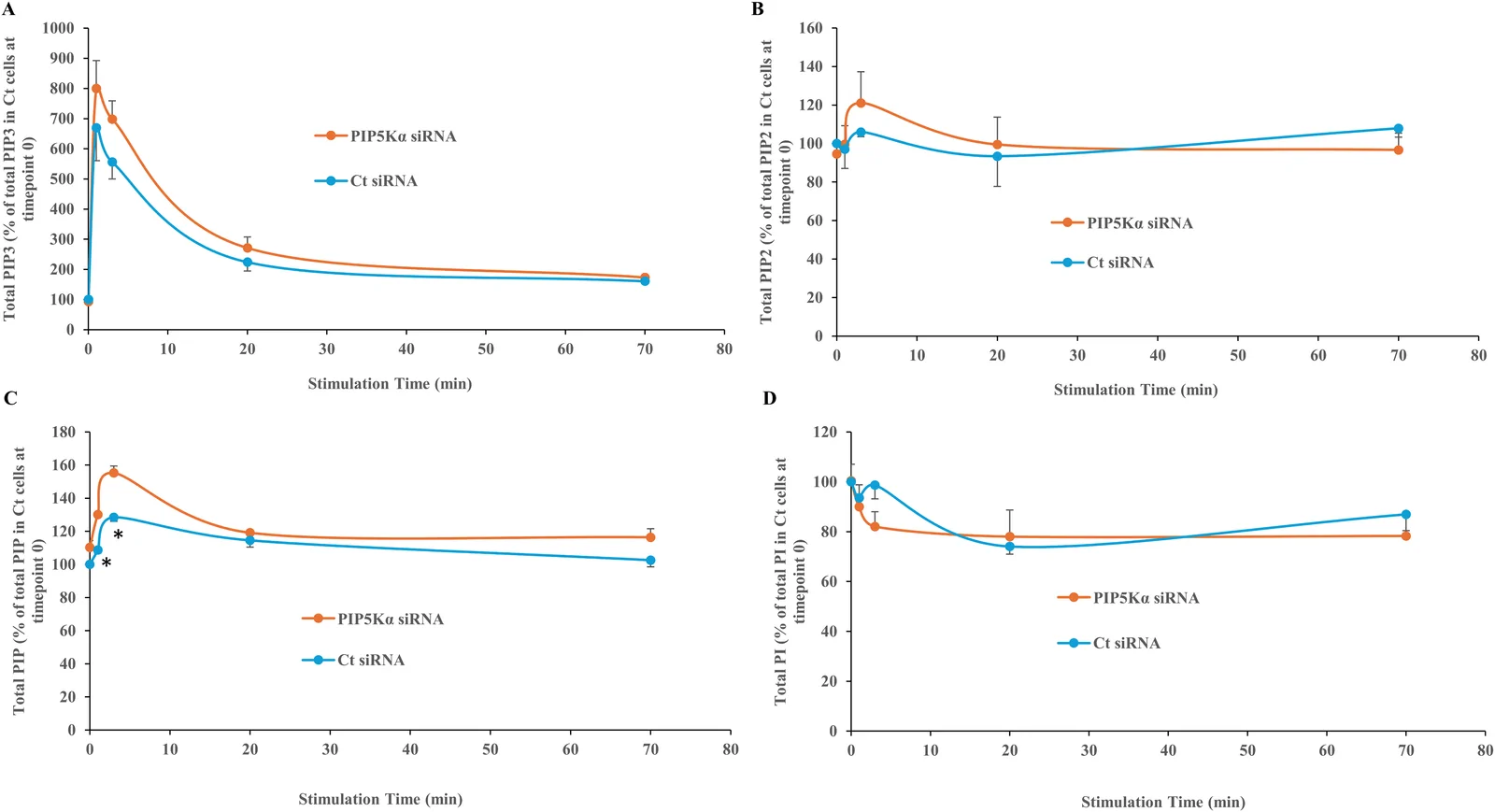
Mass spectrometry measurement of phosphoinositide molecular species after PIP5Kα siRNA suppression. A-D: After 48hr incubation with PIP5Kα- or non-targeting (control, Ct) siRNA, followed by stimulation with EGF or its vehicle, the total levels of PIP_3_, PIP_2_, PIP, and PI, including the 5 most abundant molecular species, were quantified by mass spectrometry. Recoveries were corrected by relevant ISD, normalized over total PI / ISD PI to correct for input, and are presented as % of the relevant total phosphoinositide measured in samples treated with control siRNA (Ct cells) in the absence of EGF stimulation (timepoint 0). The graphs present means and standard errors from 3 independent experiments, plotted over stimulation time in minutes. One-way ANOVA, followed by Tukey`s multiple comparison test, compared corrected normalised recoveries between different siRNA treatments for each time point. Significant result, if found, is indicated by asterisk and corresponds to p < 0.05.

**Fig 3:**
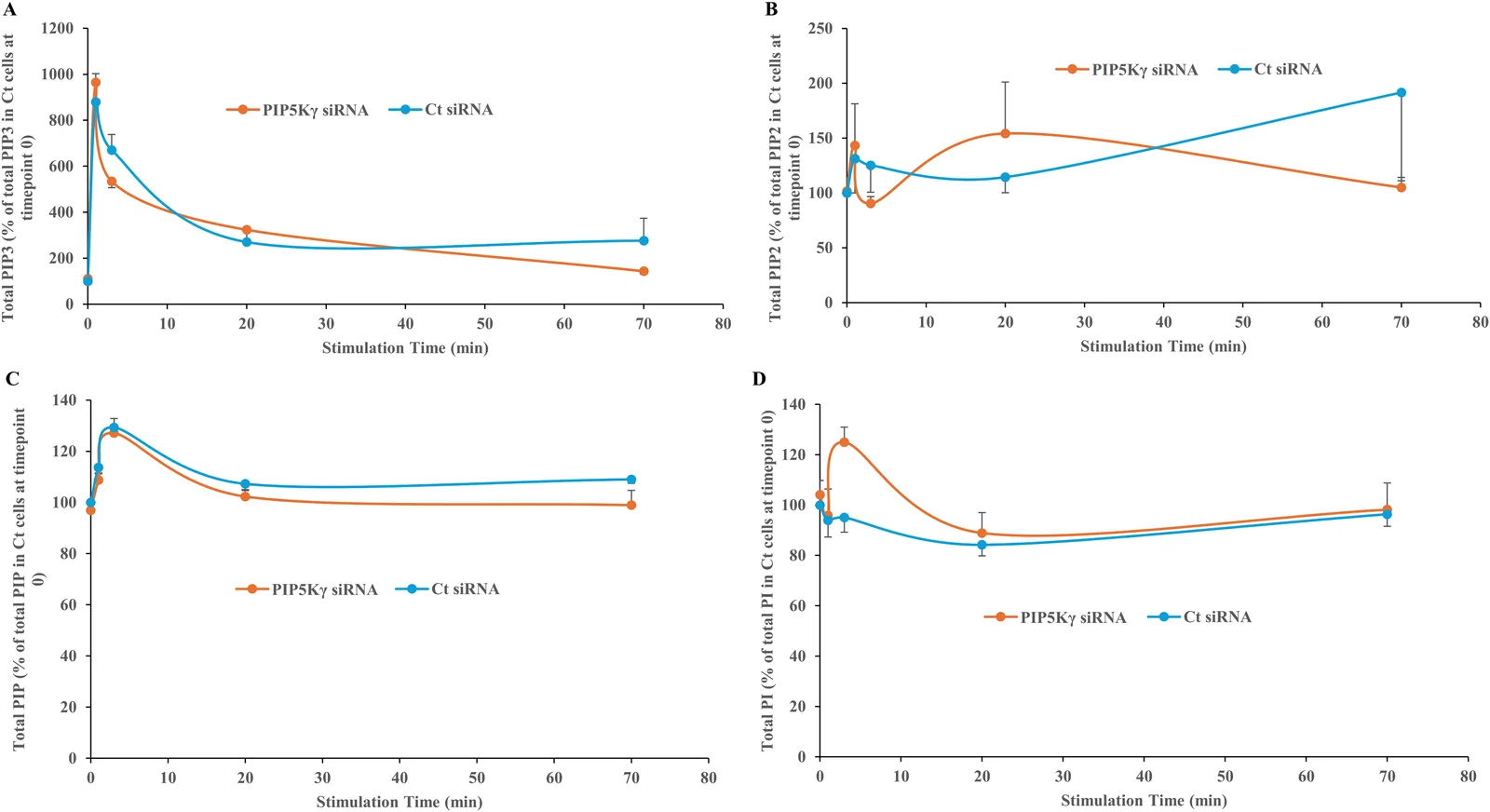
Mass spectrometry measurement of phosphoinositide molecular species after PIP5Kγ siRNA suppression. A-D: After 48hr incubation with PIP5Kγ- or non-targeting (control, Ct) siRNA, followed by stimulation with EGF or its vehicle, the total levels of PIP_3_, PIP_2_, PIP, and PI, including the 5 most abundant molecular species were quantified by mass spectrometry. Recoveries were analysed and are presented in the same way as described in Fig 2.

### Cas9/CRISPR targeting of PIP5Kα or γ in MCF10A cells

Standard Cas9/CRISPR approaches were used to target PIP5Kα or γ. Cells were transfected with a vector expressing Cas9 and the validated PIP5K-directed guides, and a puromycin-resistance vector. Puromycin-resistant cultures were generated, and cell lines were cloned by limiting dilution in 96-well plates. The cell lines were screened by western blotting for PIP5Kα or γ. MCF10A clones with undetectable or unchanged PIP5Kα or γ (transfected control, Ct), compared to parental MCF10A cells, were expanded. Cells lacking detectable PIP5Kα showed significantly increased expression of PIP5Kγ, whereas PIP5Kβ remained undetectable. Cells lacking detectable PIP5Kγ contained unchanged amounts of PIP5Kα. Again, PIP5Kβ remained undetectable (Fig 4). Successful multi-nucleotide deletion in PIP5Kα exon 1, leading to frameshift mutation and emergence of stop codons, was confirmed for PIP5Kα knockout cells (Fig 4). Multiple attempts to amplify PIP5Kγ exon 1 from PIP5Kγ knockout cells for sequencing, using different primers, were not successful. However, given the fact that all the major PIP5Kγ variants, present in wild-type cells, disappeared in PIP5Kγ knockout cells and no novel bands appeared, the possibility for a PIP5Kγ variant expression in these cells is very remote. MCF10A clones were stimulated with EGF or its vehicle and phosphoinositides were quantified by mass spectrometry. Surprisingly, there were no meaningful changes in the levels of the phosphoinositides between either the PIP5Kα or γ knockout cell lines and the ones with normal isoform expression, (transfected control and parental wild-type, Fig 5). We assume this lack of phenotype change reflects potential compensatory changes in expression or activity, leading to the observed redundancy between PIP5Kα and γ. Although, stearoyl-arachidonoyl- species (C38:4) was not the dominant one in MCF10A cells, its abundance was not altered in knockout cells compared to those with normal isoform expression. Similarly, no change was seen in the rest of the molecular species.

**Fig 4:**
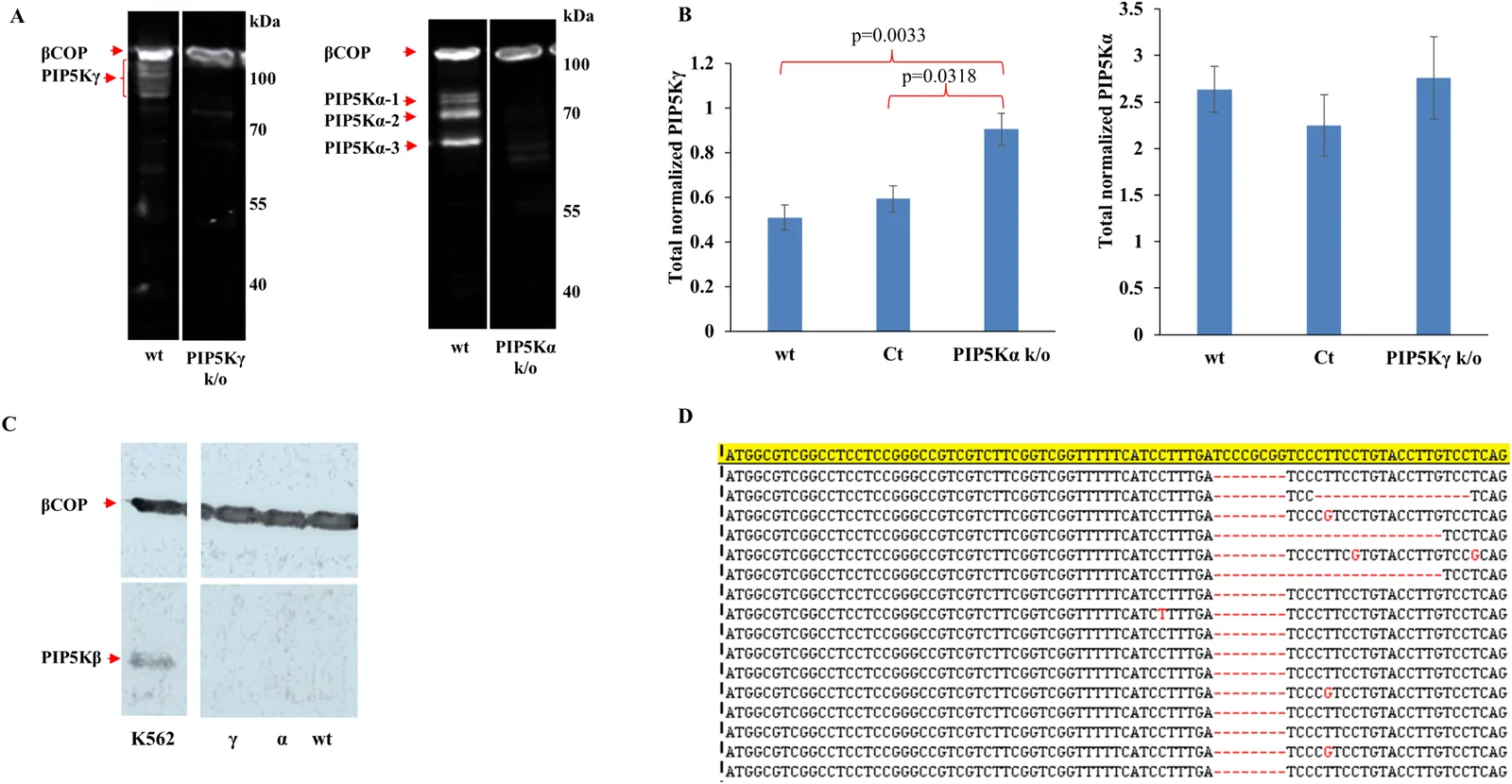
PIP5Kα or PIP5Kγ knockout clone generation from MCF10A cells by Cas9/CRISPR. A: Screening of monoclonal populations (western blotting) after targeting PIP5Kγ or PIP5Kα. A lane loaded with wild-type (wt) cell lysate (left), and one loaded with lysate lacking any detectable PIP5Kγ or PIP5Kα signal (right) from the same blot and under the same exposure are shown. βCOP was used as loading control for signal normalisation. B: Total normalized PIP5Kγ or PIP5Kα western blot signal from wt, transfected control (Ct) and PIP5Kα or PIP5Kγ knockout cells (PIP5Kα k/o, PIP5Kγ k/o) respectively. The graphs show means and standard errors from 3 independent experiments. One-way ANOVA, followed by Tukey`s multiple comparison test, compared normalised signal between wt, Ct, and either PIP5Kα k/o or PIP5Kγ k/o cells. Significant result is indicated by the p value. C: PIP5Kβ screening (western blotting) of PIP5Kγ, PIP5Kα knockout clones (γ, α) and wild-type (wt) MCF10A cells. A lane loaded with K562 cell lysate as a positive control is shown from the same blot and under the same exposure. PIP5Kβ appears as a 70 kDa band, whereas loading control (βCOP) as a 100 kDa band. D: Sequence alignments of the targeted PIP5Kα exon 1 between wild-type cells (top sequence highlighted in yellow) and PIP5Kα k/o population. The red dashes indicate deleted nucleotides, whereas insertions or replacements are shown in red font.

**Fig 5:**
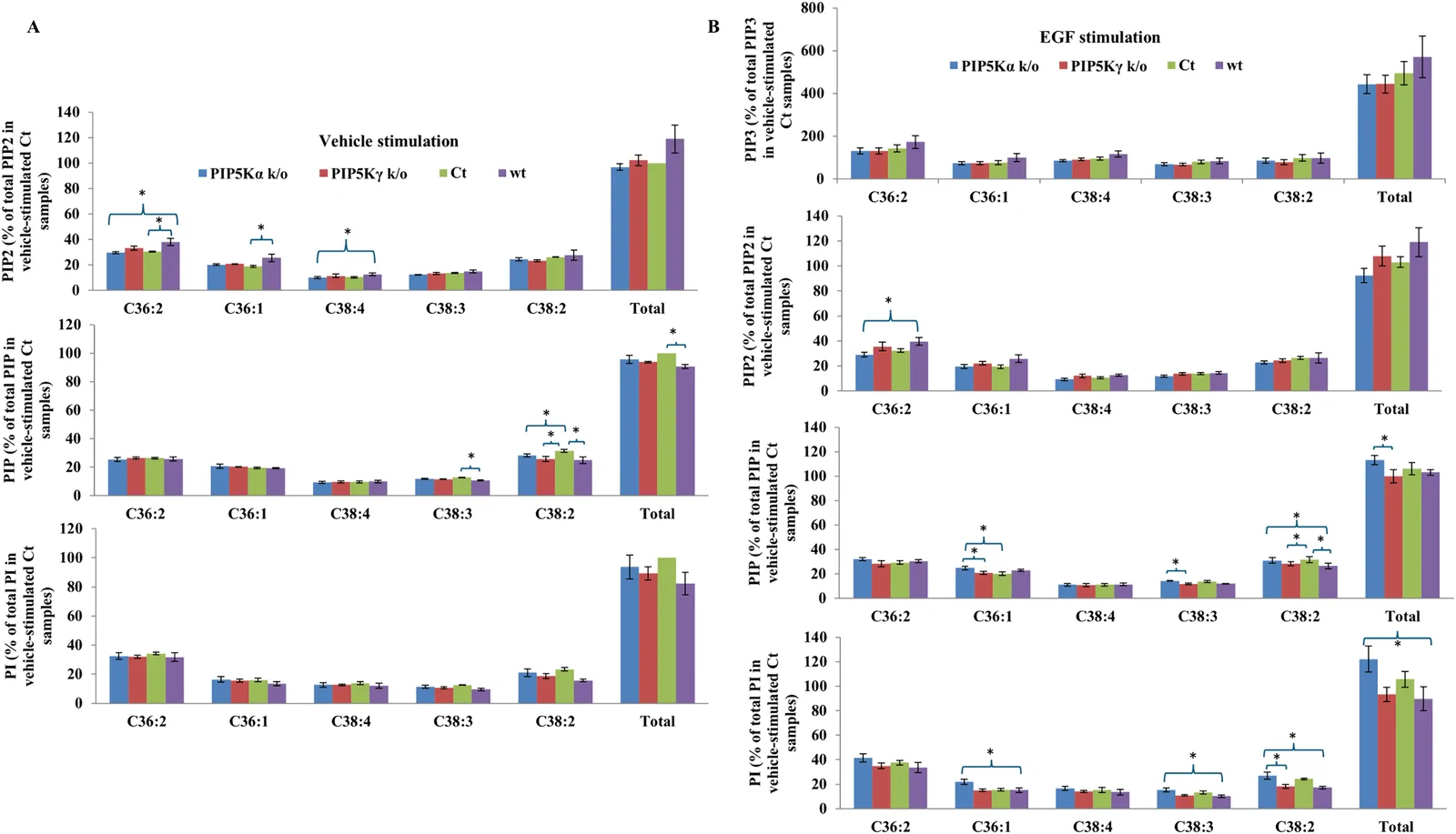
PIP5Kα or PIP5Kγ isoform knockout has no apparent effect on phosphoinositides. Monoclonal populations without any detectable expression of PIP5Kα or PIP5Kγ isoform (PIP5Kα or PIP5Kγ knockout cells, PIP5Kα k/o, PIP5Kγ k/o), transfected control cells with normal isoform expression (Ct) and parental MCF10A cells (wild-type, wt) were stimulated with EGF vehicle (A) or EGF (B) for 1 minute and the phosphoinositide molecular species were extracted and quantified by mass spectrometry. Recoveries were corrected by relevant ISD, normalized over total PI / ISD PI to correct for input and are presented as % of total lipid in Ct samples in the absence of EGF. The graphs present means and standard errors from 3 independent experiments. One-way ANOVA, followed by Tukey`s multiple comparison test, compared corrected, normalised recoveries. Significant result is represented by asterisk and corresponds to p < 0.05. No reliable quantification of PIP_3_ in the absence of EGF stimulation was possible.

### Chemical inhibition of PIP5Ks

A series of small molecule inhibitors of PIP5Ks have been described recently and demonstrated to reduce PIP_2_ synthesis in cell lines [18]. The inhibitors show good levels of selectivity for PIP5Ks compared with other kinases including PI3Kα, but are not PIP5K isoform specific. We were provided samples of two of these compounds, CRT630 and CRT349 (respectively called compounds 25 and 26 in reference 18, S5 Fig). However, the lack of any clear phenotype with PIP5Kα or γ suppression or deletion and the fact the inhibitors are not isoform specific meant we were unable to use them to infer isoform-specific roles in our results.

To test the impact of these inhibitors on PIP_2_ levels and EGF-stimulated PIP_3_ accumulation we serum-starved MCF10A cells for 16 hours, treated with inhibitors or their vehicle (1% DMSO diluted in starvation medium), stimulated with EGF or its vehicle in the continued presence of the inhibitor or its vehicle (treatment with the EGF-vehicle is referred to as “mock stimulation” in the figures to distinguish it from treatments with the inhibitor’s vehicle), before quenching the incubations and extracting and quantifying phosphoinositides by mass spectrometry. We saw no evidence that the inhibitors caused gross changes in cell adhesion or morphology, at the maximum concentrations we tested, after up to 16 hours; after 48 hours, however, a significant number of cells had detached, and the remaining adherent cells had an abnormal morphology and were rounded-up. We tested also the inhibitor vehicle (1% DMSO in starvation medium), and we found no effect on phosphoinositide recovery compared to the absence of DMSO.

Initial experiments used CRT349 at varying concentrations for 2 hours prior to 20 minutes stimulation with EGF. These experiments showed that CRT349 reduced the levels of PIP_2_ in a dose-dependent fashion. There was a parallel increase in PIP levels. EGF-stimulated accumulation of PIP_3_ was also substantially inhibited.

In further experiments we reduced the time for which the cells were exposed to the inhibitor to 30 minutes, to further mitigate against potentially indirect effects of the inhibitors including potential compensatory signalling and/or “rewiring”. The time of stimulation with EGF was also reduced to 1 minute to maximise the PIP_3_ signal. It is important to note that PIP_3_ levels in the absence of EGF were indistinguishable from machine noise. In this setting, total PIP_2_ levels were reduced by the inhibitor with an apparent IC50 of 30 µM, while a parallel increase in total PIP levels was observed (Fig 6). EGF-stimulated PIP_3_ accumulation was inhibited with an apparent IC50 of 2 µM (Fig 6). These results support the idea that there is a pool of PIP_2_, preferentially used by class I PI3K signalling for PIP_3_ production and more sensitive to the PIP5K inhibitor. After using a non-linear regression log (inhibitor) vs response model and comparing IC50 between molecular species, we found that the proportions of none of the individual molecular species variants of any of the phosphoinositides we measured were significantly changed by the inhibitor in either the presence or absence of EGF (S6 and S7 Figs). Broadly the impact of the inhibitor was very similar to that seen in the pilot experiments in which the inhibitor was pre-incubated with the cells for 2 hours and the cells were stimulated with EGF for 20 minutes, suggesting these effects are not particularly sensitive to these parameters.

**Fig 6:**
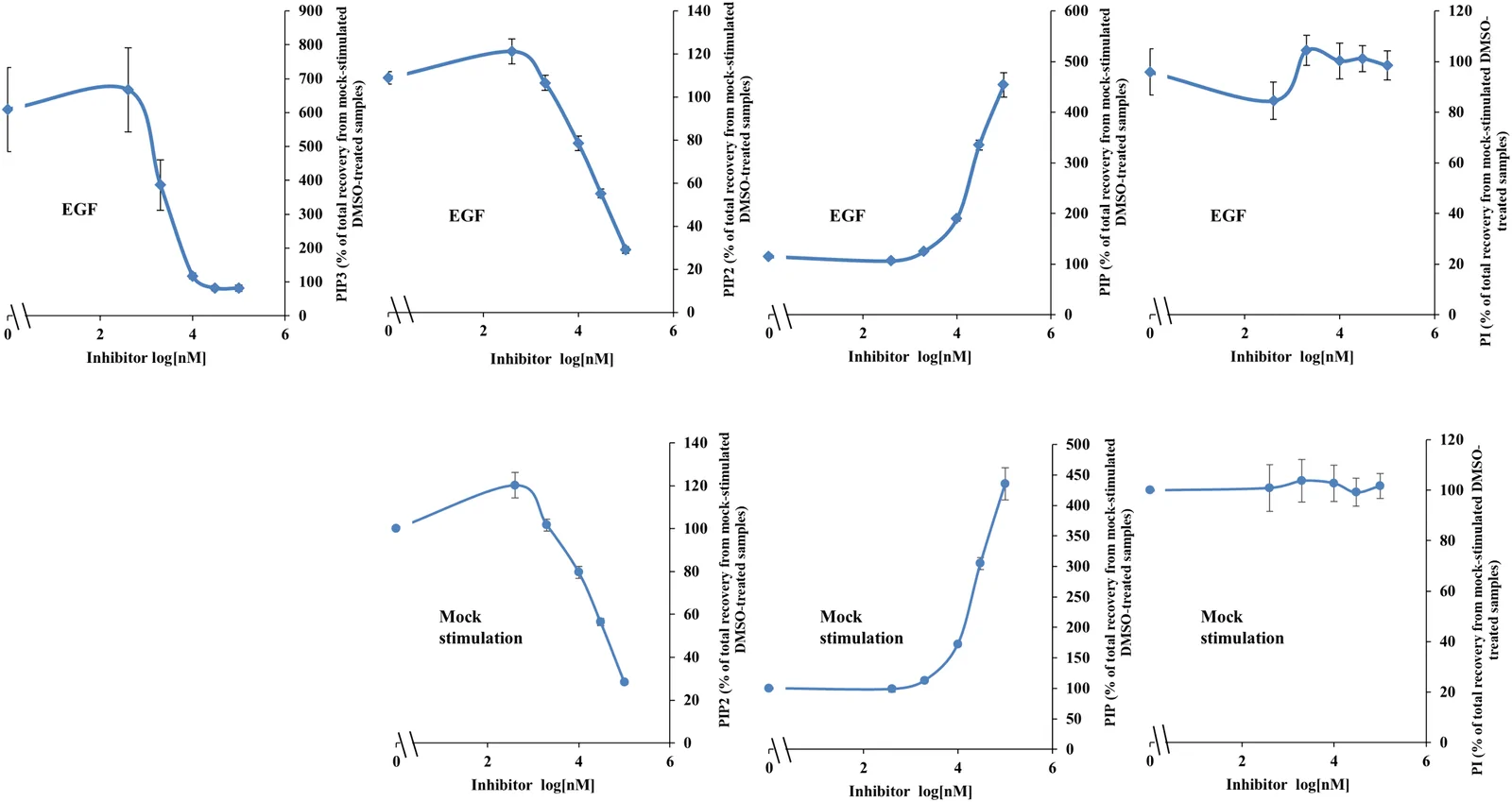
PIP5K chemical inhibition by CRT349. Mass spectrometry measurement of total PIP_3_, PIP_2_, PIP, and PI after 30-minute incubation of MCF10A cells with varying concentrations of the PIP5K inhibitor CRT349 or its vehicle (DMSO), followed by stimulation with EGF or its vehicle (mock stimulation) for 1 minute. Mass spectrometry recoveries were corrected over relevant ISD and normalized over total PI / ISD PI to correct for cell input. They are plotted as % of total recoveries from mock-stimulated DMSO-treated samples against inhibitor concentrations in nanomolar, shown in logarithmic scale (log[nM]). The gap on the horizontal axis separates the logarithmic scale from 0 nM of inhibitor (DMSO treatment). The graphs show means and standard errors from 6 independent experiments.

Phospho-308-AKT (P308-AKT) and phospho-473-AKT (P473-AKT) were quantified in the same experiments by immuno-blotting, and this revealed that EGF-stimulated AKT phosphorylation responded to the PIP5K inhibitor similarly to the EGF-stimulated PIP_3_ accumulation, indicating that the cell’s PIP_3_-sensitive effectors were perceiving a similar decrease in PIP_3_ to that we had measured (Fig 7A). In order to investigate whether the compound affected the activation of EGF receptors, both the total receptor protein (EGFR) and the EGF receptor, phosphorylated at Tyr 1068 (p-EGFR) were quantified. Tyr 1068 is an auto-phosphorylation site that becomes phosphorylated following agonist binding, and is important for Grb2 binding, Ras and Mitogen-activated protein kinase (MAPK) activation. CRT349 was able to cause a dose-dependent reduction in p-EGFR but the compound did not affect total EGFR (Fig 7B). These results suggested CRT349 can inhibit (either directly or indirectly) EGFR tyrosine kinase activation. Interestingly, in EGF-stimulated samples, the sensitivity of EGFR phosphorylation to CRT349 was most similar to that of PIP_2_. This would be consistent with a report that EGFR activation is dependent on PIP_2_ binding to a small motif in its juxta-membrane domain [39]. These results suggest that signalling downstream of the EGFR might be broadly inhibited, i.e., more broadly than just class I PI3K signalling. To test that idea, we decided to assay EGF-stimulated MAPK activation by monitoring the phosphorylation of MAPK (p-MAPK, MAPK1, phosphorylated at Thr 202 and Tyr 204 or at Tyr 204 only, as well as MAPK2, phosphorylated at Thr 185 and Tyr 187 or at Tyr 187 only) by immuno-blotting. The data showed that CRT349 could inhibit EGF-stimulated phosphorylation of MAPK (Fig 7C). Activation of MAPK can be either dependent or independent of class I PI3K activity in different cell lines.

**Fig 7:**
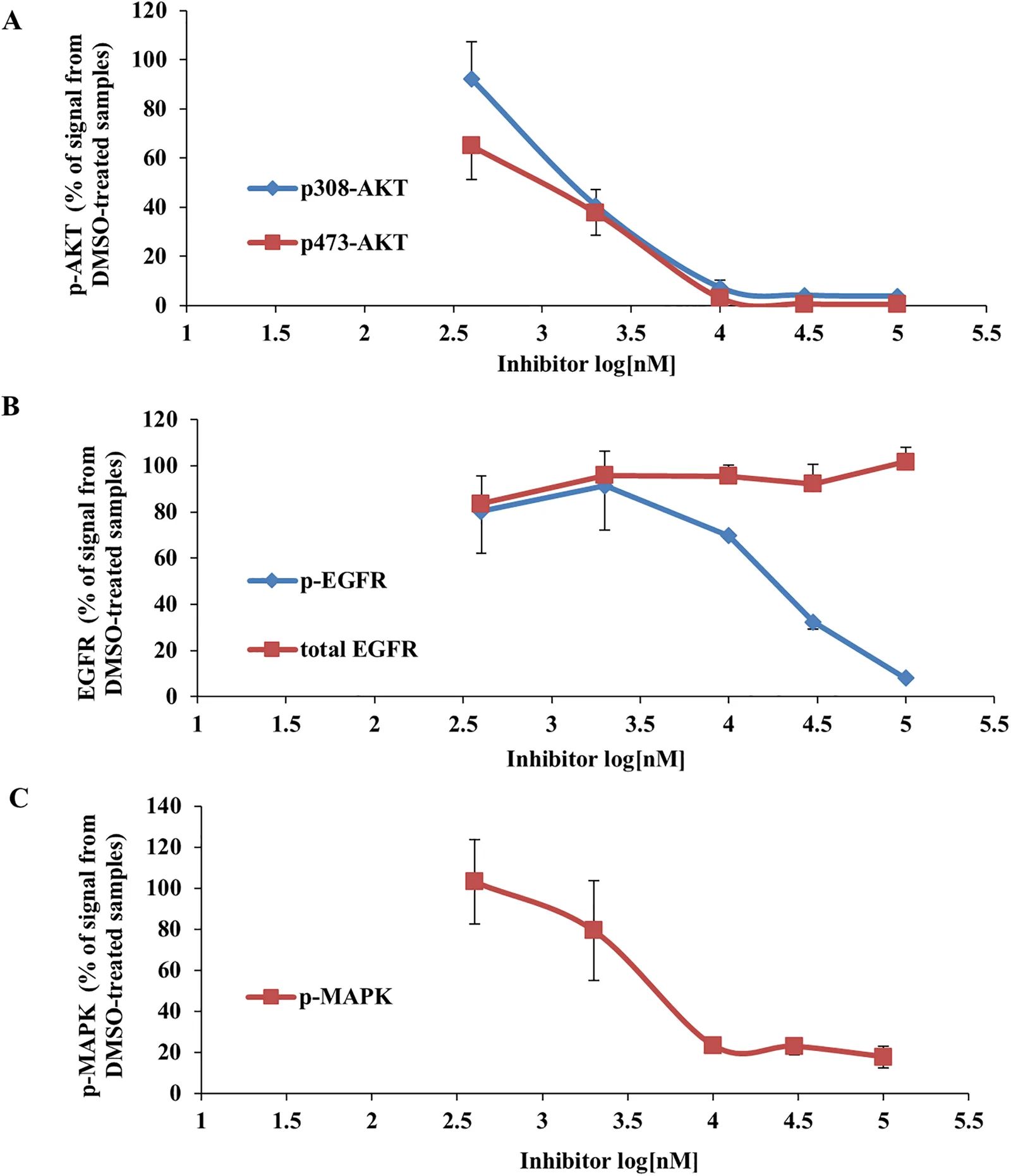
The PIP5K inhibitor CRT349 reduces EGF-dependent phosphorylation of AKT, EGFR, and MAPK. A-C: MCF10A cells were incubated with varying concentrations of the PIP5K inhibitor CRT349 or its vehicle (DMSO) for 30 minutes, followed by 1-minute stimulation with EGF. Cell lysates were analysed (western blotting) for phospho-308-AKT (p308-AKT), phospho-473-AKT (p473-AKT), phosphorylated EGFR (p-EGFR), total EGFR, and phosphorylated MAPK (p-MAPK). Fluorescent signals were corrected over loading control (βCOP) and are plotted as % of respective signal from control samples, treated with DMSO against inhibitor concentrations in nanomolar, shown in logarithmic scale (log[nM]). The graphs show means and standard errors from 3 independent experiments.

To understand the relationship between MAPK and class I PI3K activation in our data, we pre-treated EGF-stimulated or control MCF10A cells with a potent PI3Kα-selective inhibitor A66 (EGF-stimulated PIP_3_ accumulation is preferentially mediated by PI3Kα). Initial experiments showed that A66 inhibited accumulation of PIP_3_ and phosphorylation of AKT but had no effect on total EGFR or EGFR phosphorylation (S8 Fig). Moreover, A66 reduced EGF-stimulated MAPK phosphorylation (S8 Fig), indicating some level of cross-talk between these pathways and that the ability of PIP5K inhibitors to reduce MAPK phosphorylation may be at least partially a result of inhibition of PIP_3_ accumulation and may not be totally independent.

Collectively, the immuno-blotting data show that p-AKT followed exactly the same pattern of changes as PIP_3_. Taken together the data suggest that the inhibitor affected PIP5K activity, as readout by the increase in PIP and decrease in PIP_2_ and EGFR activation, leading to an impairment of EGF signalling, including both class I PI3K and MAPK activation. The simplest explanation is that the reduction in PIP_3_ accumulation is a result of both reduced levels of PIP_2_-substrate and inhibition of EGFR signalling more broadly. The cause/effect relationships between these events remained, at this point, very unclear and could have resulted from some being the result of off-target effects of the inhibitor. Hence, the initial priority was to assess whether any of these results were the outcome of random off-target activity by using a distinct but similar small molecule PIP5K inhibitor, and whether CRT630 might be a better tool compound in cell-based assays. To achieve this, we performed experiments similar to those in which we tested CRT349 to investigate its impact on the levels of phosphoinositides, p-AKT, p-MAPK, p-EGFR and total EGFR. In these experiments with CRT630 we focused on 30-minute pre-incubation with the inhibitor or its vehicle and 1-minute stimulation with EGF or its vehicle (mock stimulation). CRT630 was used at the same concentrations as CRT349, however, because the compound formed visible precipitate in solutions at 100 μM CRT630, the maximum concentration was 30 μΜ in these assays. Those experiments revealed that CRT630 significantly; reduced PIP_2_ levels, increased PIP levels and reduced EGF-stimulated accumulation of PIP_3_ without affecting PI levels at increasing concentrations up to 10 μM (Fig 8A). At 30 μM, the dose-response curves either reached a plateau or showed a completely different and unexpected trend (decrease in PIP and increase in PI). However, it is possible that the compound might have reached its solubility limit at 30 μM (visible precipitations were seen at 100 μM), thus causing issues of variable cellular uptake and nonspecific effects. None of the molecular species of any of the phosphoinositides measured in these experiments were significantly differentially impacted by either EGF or CRT630 (S9 and S10 Figs), as shown by a non-linear regression log (inhibitor) vs response analysis, comparing IC50 values. Furthermore, CRT630 significantly; reduced AKT phosphorylation, reduced MAPK phosphorylation and EGFR phosphorylation but had no effect on total EGFR (Fig 8B). These results are very similar in pattern to those obtained with CRT349. Notably, the IC50 values for PIP5K activity, observed in our cell-based assay using MCF10A cells, were at least a thousand times higher than the IC50 for PIP5K activity seen with *in vitro* recombinant protein assays (S5 Fig). Both compounds reduced PIP_3_ & AKT phosphorylation with an IC50 ~ 2 µM in our cell assay. That is well below the IC50 observed using recombinant PI3K or AKT, suggesting that these effects were not from direct PI3K or AKT compound inhibition. CRT630 reduced MAPK phosphorylation with a similar IC50 in our cell assay (~2 µM). Given the IC50 using recombinant MAPK (S5 Fig), we suggest that the reduction in MAPK phosphorylation in MCF10A cells was not primarily caused by direct MAPK inhibition by the compound. In terms of EGFR phosphorylation, the CRT630 IC50 was only twelve times higher for EGFR than PIP5Kα in recombinant protein assays, and, although no data from recombinant EGFR assays exists for CRT349, similar fold difference between EGFR and PIP5K selectivity is expected for this compound due to close structural similarity. Therefore, we cannot exclude the possibility of some direct compound inhibition on EGFR phosphorylation especially at higher concentrations.

**Fig 8:**
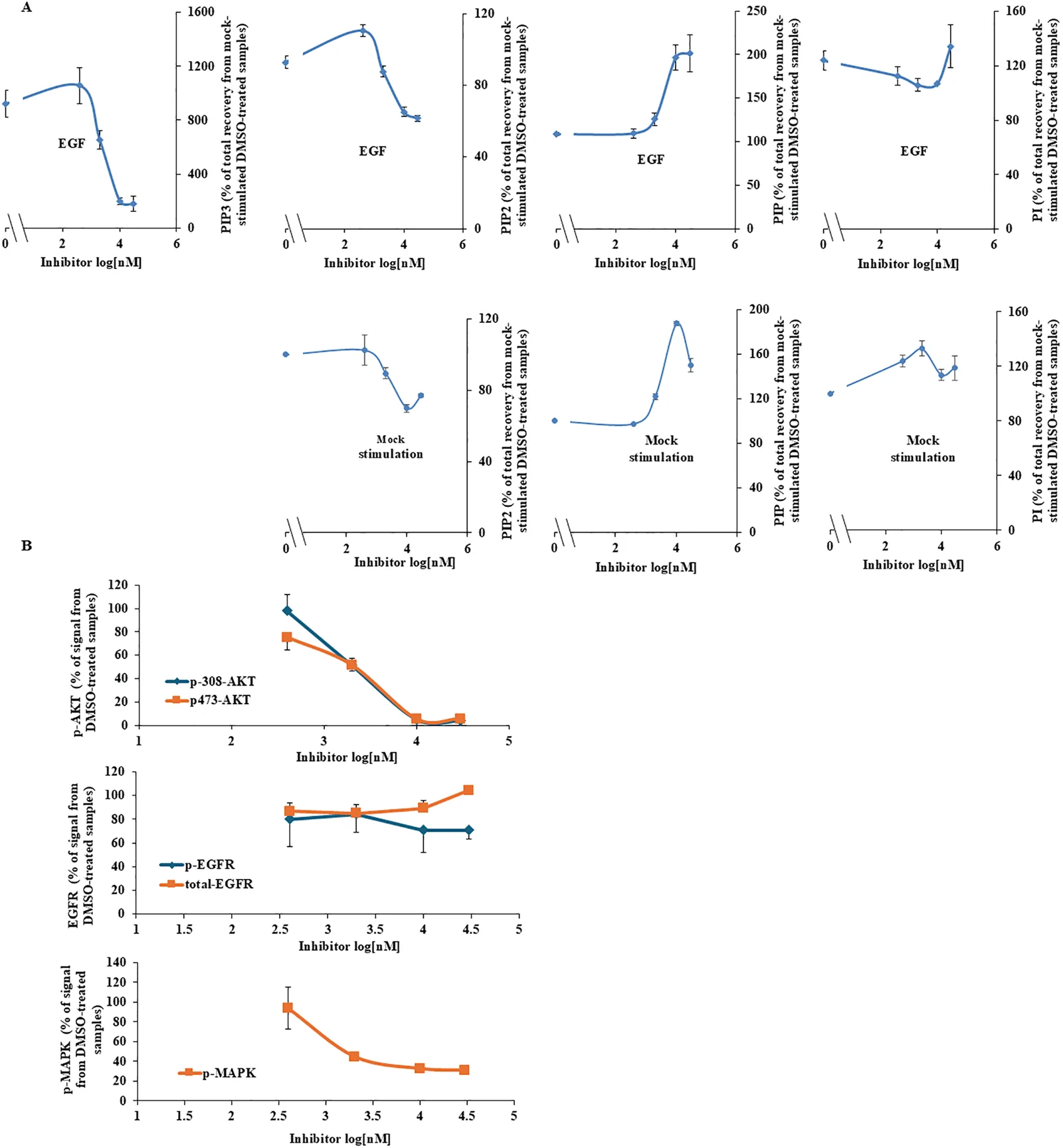
PIP5K 30-minute chemical inhibition by CRT630. A: Mass spectrometry measurement of total PIP_3_, PIP_2_, PIP, and PI after 30-minute incubation of MCF10A cells with varying concentrations of the PIP5K inhibitor CRT630 or its vehicle (DMSO), followed by stimulation with EGF or its vehicle (mock stimulation) for 1 minute. Mass spectrometry recoveries were corrected and presented as explained in Fig 6. The graphs show means and standard errors from 3 independent experiments. B: A portion of same samples was analysed (western blotting) for phospho-308-AKT (p308-AKT), phospho-473-AKT (p473-AKT), phosphorylated EGFR (p-EGFR), total EGFR, and phosphorylated MAPK (p-MAPK). Fluorescent signals were corrected and presented as explained in Fig 7. The graphs show means and standard errors from 3 independent experiments.

### Chemical inhibition of PIP5K activity in k/o cell lines

To investigate whether PIP5Kα or PIP5Kγ plays a more important role either in the total PIP_2_ production and/or in PI3K signalling, parental (wild-type, wt) and k/o cell lines were incubated for 30 minutes with the same range of CRT349 concentrations, used before, or its vehicle (DMSO) before stimulation with EGF or its vehicle (mock stimulation) for 1 minute. We hypothesised that acute inhibition of the single remaining PIP5K isoform in the k/o cell lines could reveal differences in the importance of each isoform by affecting the cellular phosphoinositides to different extent. As seen previously, a dose-dependent reduction in total PIP_3_ and PIP_2_ levels and increase in total PIP was observed in all cell lines, whereas total PI was not affected (Fig 9). Within each cell population, the pattern of changes was the same between the molecular species (S11 and S12 Figs). Non-linear regression (log (inhibitor) vs response) and curve fit comparison analysis revealed much higher sensitivity of PIP_3_, PIP_2_, and PIP in the PIP5Kα k/o cell line compared to the PIP5Kγ k/o one, with the apparent IC50 values for PIP_3_ and PIP_2_ being 3- and 13-fold lower respectively (Fig 9). The analysis, however, did not give any evidence for a possible difference in the importance of PIP5Kα or PIP5Kγ for total PIP_3_ and/or PIP_2_ production. The higher sensitivity in PIP5Kα k/o cells could be explained by the presence of PIP5Kγ, as the only PIP5K isoform, that is more potently inhibited by the compound. *In vitro* assays revealed that the IC50 of PIP5Kγ is at least 11-fold lower than PIP5Kα [18]. It should also be pointed out that in these assays the apparent IC50 values for PIP_2_ and PIP_3_ differ slightly from the ones obtained in previous experiments with wt cells (Fig 6). However, different batches of the compound, used in these experiments, may account for these differences. As seen before, the reduction in PIP_3_ was much more efficient than in PIP_2_ in wt and PIP5Kγ k/o cells, suggesting the possible presence of a PI3K-dedicated PIP_2_ pool which is more sensitive to chemical inhibition. However, in PIP5Kα k/o cells, PIP_2_ and PIP_3_ were reduced with similar efficiency, suggesting this possible distinction between two pools of PIP_2_ is not decisive.

**Fig 9:**
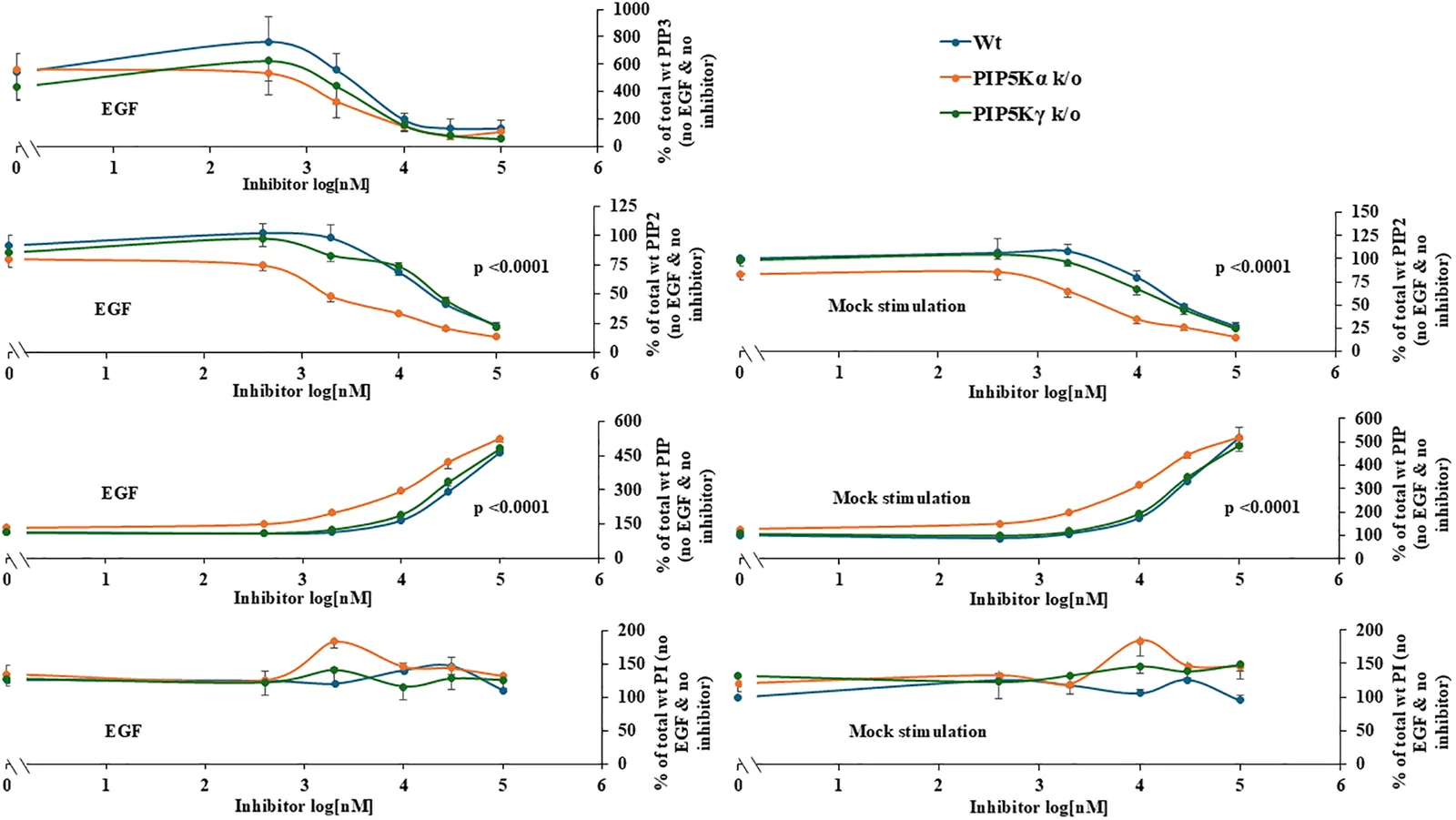
PIP5K 30-minute chemical inhibition by CRT349 in PIP5K knockout cell lines. Mass spectrometry measurement of total PIP_3_, PIP_2_, PIP, and PI after 30-minute incubation of parental (wild-type, wt), PIP5Kα or PIP5Kγ knockout (PIP5Kα k/o, PIP5Kγ k/o) MCF10A cells with varying concentrations of the PIP5K inhibitor CRT349 or its vehicle (DMSO), followed by stimulation with EGF or its vehicle (mock stimulation) for 1 minute. Mass spectrometry PIP_3_, PIP_2_, and PIP recoveries were corrected over relevant ISD and normalized over total PI / ISD PI to correct for cell input. They are plotted as % of total recoveries from mock-stimulated wild-type samples, treated with DMSO, against inhibitor concentrations (nM), shown in logarithmic scale. The graphs show means and standard errors from 3 independent experiments. The gap on the horizontal axis separates the logarithmic scale from 0 nM of inhibitor (DMSO treatment). P values, if displayed, show significant result, found by F test.

## Discussion

There is a large body of data that shows EGF can stimulate PLC activation in cells [40,41], but in the large majority of cases these results were obtained in cells over expressing EGF receptors. We find that endogenous EGFR on non-transformed MCF10A cells cannot significantly activate PLC signalling in parallel with activation of class I PI3Ks. Furthermore, ligands, such as ATP, that were capable of stimulating increases in intracellular free Ca^2+^ in MCF10A cells, could not activate class I PI3K signalling. These observations limited our opportunity to study the role of PIP5Ks in generation of PIP_2_ pools that might be selectively involved in supply of PLCs and class I PI3Ks because, as soon as different agonists are used to activate PLC or class I PI3K signalling, any differences could be related to agonist-related differences.

Our data suggest MCF10A cells only express PIP5Ks α and γ at both mRNA and protein levels. Both variants could be detected as multiple, PIP5K-knock-down-sensitive bands in immune-blots. The spread of sizes suggests the variants are unlikely to be solely a result of distinct phosphorylated states. It remains possible they are a result of differential RNA splicing [42–44] and/or ubiquitinylation [45] but this was not explored.

In this study we have applied a methodology that cannot distinguish between the different regio-isomers of PIP (PI4P, PI3P, PI5P in order of abundance) and PIP_2_ (PI(4,5)P_2_, PI(3,4)P_2_ and PI(3,5)P_2_ in order of abundance). Hence, we measured the sum of PIP and PIP_2_ regio-isomers (over 90% PI4P and PI(4,5)P_2_ respectively). Lipidomic methods have been developed using high resolution chromatography to resolve some, or all, of these regio-isomers [38,46] but were not applied here.

RNAi suppression of PIP5Ks (with typically 70–75% reductions in target) did not substantially alter the acyl-chain profile of any of the phosphoinositides in either basal or EGF-stimulated MCF10A cells. Thus, we find no evidence that in MCF10A cells specific PIP5K isoforms are dedicated to production of specific (e.g., hormone-sensitive) pools of PIP_2_ and/or PIP_3_. Previous work has indicated that PIP5Ks can display a preference for stearoyl/arachidonoyl-substrates *in vitro* [23]. Recent work that has profiled the molecular species of phosphoinositides in hormone-stimulated and control platelets and cancer cell lines [27,47], however, did not observe preferential resynthesis of stearoyl/arachidonoyl-PIP_2_; also indicating that in those contexts the relevant PIP5Ks do not display acyl-chain selectivity *in vivo*. These conclusions are supported by the lack of impact of the PIP5K inhibitors on the acyl-chain composition of PIP_2_ and PIP_3_.

RNAi suppression of PIP5Kα (with a concomitant increase in PIP5Kγ levels) led to a very small increase in the levels of PIP and PIP_3_ in MCF10A cells. Suppression of PIP5Kγ was without effect. Combined suppression also had no significant effect on poly-phosphoinositides but led to a large increase in the variance of our measurements as a result of an unexpected decrease in PI levels and a change in data normalisation. We do not understand why the levels of PI fell as the cells appeared viable within the time scale of the experiments. These results are inconclusive and it remains possible that PIP5Ks have isoform-specific roles in synthesis of functionally discrete pools of PI(4,5)P_2_. The truth could be masked by a combination of problems, including partial suppression and weak isoform expression, adaptive compensation by increased expression of the other isoform (increased expression of PIP5Kγ after PIP5Kα suppression), and / or other potential adaptive compensation mechanisms, affecting phosphoinositide metabolism, to maintain the endogenous phosphoinositide levels after several days of RNAi-suppression. We attempted to deconvolve some of these issues by creation of stable PIP5Kα and γ k/o clones using gene editing and a new series of rapid-acting, small molecule PIP5K-selective inhibitors.

We successfully deleted PIP5Kα or γ from MCF10A cells and immuno-blotting indicated the targeting strategy had not been circumvented. Compared with normal expression of two PIP5K isoforms in control and wild-type cells, the presence of one isoform in knockout cells did not alter the phosphoinositide levels or their acyl-chain profile. It is not clear whether this observed redundancy between PIP5Kα and PIP5Kγ is physiologically relevant or whether it derives from adaptive compensatory changes in enzyme expression or activity. Notably, as seen with the siRNA suppression of PIP5Kα, the deletion of PIP5Kα led to an increased expression of the remaining PIP5Kγ.

Cells subjected to genetic methods of enzyme depletion have substantial time to adapt and activate compensatory mechanisms, leading to the observed absence of phenotype after either siRNA suppression or CRISPR-Cas9 deletion of one PIP5K isoform. Therefore, it is not clear whether the apparent PIP5K isoform redundancy is physiologically relevant in MCF10A cells. We attempted to exclude the effect of cellular adaptation and compensatory mechanisms by acute PIP5K inhibition using chemical compounds, minimising the incubation time. The small molecule inhibitors proved to be well tolerated by the MCF10A cells and, importantly, both inhibitors rapidly and substantially depleted PIP_2_ levels. The compounds showed evidence of isoform selectivity by the difference in their apparent IC50 for PIP_2_ depletion in different PIP5K k/o cell lines compared to wild-type cells. However, at the doses we applied to wild-type cells the compounds were not sufficiently selective to be capable of isolating PIP5Kα from PIP5Kγ function. Nevertheless, the inhibitors efficiently reduced PIP_2_ levels unlike RNAi or k/o strategies. The simplest explanation is that the genetic methods were limited by compensation mechanisms, probably acting at the level of the specific catalytic activity of the kinases rather than their expression, that became manifest during the 48hr (or longer in the case of the k/o) course of the experiments. We do not know what happens to PIP_2_ levels in cells treated with PIP5K inhibitors for similarly extended periods as it was clear cell adhesion/morphology became substantially compromised. Hence it is unclear whether the compensation, that tends to increase PIP_2_ is triggered by reduced PIP_2_ levels or by reduced PIP5K protein/mRNA.

Interestingly, the PIP5K inhibitors caused a rapid and substantial increase in PIP either in the presence or absence of EGF in MCF10A cells. We assumed this is PI4P but have no evidence to confirm that, since our lipidomic approach could not distinguish between PI3P, PI4P, and PI5P. The molecular explanation for, or location of the increase is not clear. We assumed that in the unstimulated state, at least, the cells and phosphoinositide metabolism were at steady state. For the levels of PIP to increase as a sole result of reduced consumption due to the inhibitor could imply that the rate of phosphorylation of PIP by PIP5Ks is the primary determinant of its levels that cannot be compensated by changes in PI kinase and PIP/PIP_2_ phosphatase activity (assuming the decrease in PIP_2_ would result in a decrease in PIP_2_ phosphatase activity). This has some similarities to how the levels of PI5P are thought to be determined by PIP4K activity [48] but would, nevertheless, be very unlikely given the relatively high levels and active turn-over of the major PIP species, PI4P. Therefore, assuming that the observed dose-dependent increase in PIP derived mainly from PI4P accumulation, the reduced consumption of PIP because of PIP5K inhibition, combined with enhanced PI kinase activity and / or complicated activity changes in phosphoinositide phosphatases could explain this observation. Alternatively, but not exclusively, a mechanism such as de-repression of “product” feedback by PIP_2_ at the level of PI kinases could be a plausible explanation. There is evidence that PI(4,5)P_2_ can inhibit PIP5Ks at relatively high concentrations [13,14]. This is particularly reasonable given evidence of the known ability of cells to defend their PIP_2_ levels in the face of dramatic changes in consumption and a need to maintain PIP/PIP_2_ levels in the plasma membrane to sustain signalling by PLCs/PI3Ks and maintain chemical gradients for a range of lipid transport processes [49].

In MCF10A cells, we found clear evidence that the PIP5K inhibitors reduced EGF-stimulated PIP_3_ accumulation and AKT phosphorylation in unison in-line with our initial working hypothesis, however, we also found reduced EGFR phosphorylation and MAPK activation. Although a direct off-target compound inhibition in EGFR cannot be excluded (CRT630 IC50 was only twelve times higher for EGFR than PIP5Kα in recombinant protein assays), it still remains possible that inhibitor-induced reductions in PIP_2_ lowered EGFR activation [39] given the striking similarity in the dose-dependent reduction of PIP_2_ and EGFR phosphorylation by CRT349. The reduced EGFR activation, combined with a reduction in PIP_2_-substrate for PI3Ks, resulted in reduced PIP_3_ and AKT phosphorylation. The ability of PI3K-selective inhibitors to suppress EGF-stimulated MAPK activation, as indicated by preliminary experimental work, suggested that the reduction in MAPK activation probably resulted from a combination of lower PIP_3_ accumulation as well as reduced direct activation Ras/MAPK flowing from weaker activation of the EGFR.

## Conclusion

We conclude that PIP5Ks generate a substantial proportion of PIP_2_ in MCF10A cells. We found, however, no evidence for either a unique, long-term (“genetic”) role for either PIP5Kα or γ in supporting basal or EGF-stimulated PIP_2_ or PIP_3_ levels nor a role for PIP5Ks in shaping the acyl-chain profile of PIP_2_ in this cell line. We found evidence that acute inhibition of MCF10A PIP5Ks reduced EGF-stimulated PIP_3_ accumulation by reducing EGFR activation and, presumably, by depletion of the PIP_2_ substrate of PI3Ks. The difference between genetic and acute inhibition of PIP5Ks and the rapid increase in PIP upon acute inhibition of PIP5Ks suggests homeostatic mechanisms compensate changes in PIP_2_ levels.

## Supporting information

S1 Fig ATP but not EGF is a possible activator of PLC pathway, while EGF and insulin but not ATP can stimulate PI3K in MCF10A cells. A, B: MCF10A cells were mock-stimulated with imaging buffer at 30 seconds, with 10 or 100 ng/ml EGF at the end of first minute and with 10 or 100 μΜ ATP at the end of eleventh minute. Measurable Ca^2+^ responses were recorded only after ATP stimulation. Each graph corresponds to a representative sample (A: n = 4, B: n = 2). C: Stearoyl-arachidonoyl-DAG was measured after stimulation with EGF or its vehicle for 15 or 30 seconds (15 s, 30 s), 1 minute or 2 minutes (1 min, 2 min) by mass spectrometry. Recoveries were corrected over relevant internal standard (ISD) and are presented as % of respective values in vehicle-stimulated samples (control, Ct). The data shown are means plus or minus the range (n = 2–4). D: After radiolabelling of MCF10A cells with [^3^H]-inositol, [^3^H]-InsP_3_ was measured (dpm) following stimulation for 30 seconds or 1 minute (30 s, 60 s) with EGF or its vehicle (control, Ct). The columns present the average dpm values of 2 replicate samples plus or minus the range. E: Mass spectrometry measurement of C36:2, C36:1, C38:4, C38:3, and C38:2 PIP3 species, corrected for recovery via the relevant ISD and cell input by normalisation to the relevant molecular species of PI / ISD PI, in MCF10A cells after stimulation with 10 μM ATP or its vehicle (control, Ct) for 30 seconds. Stimulation with 10 ng/ml EGF for 1 minute was used as positive control. The columns present mean values from three independent experiments and standard errors. F: MCF10A cells were mock-stimulated with imaging buffer at 30 seconds, 100 μg/ml insulin at the end of first minute and 10 μΜ ATP at the end of eleventh minute. Measurable Ca^2+^ responses were recorded only after ATP stimulation. The graph shows data from 1 representative sample (n = 4).
(TIF)

S2 Fig PIP5Kα or PIP5Kγ siRNA suppression reduces all variants similarly to the total isoform. A, B: After single transfection and 48hr incubation with PIP5Kα-, PIP5Kγ- or non-targeting (control, Ct) siRNA, specific western blot bands were normalized over loading control (βCOP) and quantified as % of respective normalized signal in Ct siRNA samples. The graphs show means and standard errors from 3 independent experiments. One-way ANOVA, followed by Tukey`s multiple comparison test, compared normalised band signal between individual variants. No significant results were confirmed.
(TIF)

S3 Fig Mass spectrometry measurement of phosphoinositide molecular species after PIP5Kα siRNA suppression. A-D: After 48hr incubation with PIP5Kα- or non-targeting (control, Ct) siRNA, followed by stimulation with EGF or its vehicle, the levels of the 5 most abundant PIP_3_, PIP_2_, PIP, and PI molecular species were quantified by mass spectrometry. Results for each individual molecular species and the total phosphoinositides are presented in the same way as described in Fig 2 (means and standard errors from 3 independent experiments, plotted over stimulation time in minutes). One-way ANOVA, followed by Tukey`s multiple comparison test, compared corrected normalised recoveries between different siRNA treatments for each time point. Significant result, if found, is indicated by asterisk and corresponds to p < 0.05.
(TIF)

S4 Fig Mass spectrometry measurement of phosphoinositide molecular species after PIP5Kγ siRNA suppression. A-D: After 48hr incubation with PIP5Kγ- or non-targeting (control, Ct) siRNA, followed by stimulation with EGF or its vehicle, the levels of the 5 most abundant PIP_3_, PIP_2_, PIP, and PI molecular species were quantified by mass spectrometry. Results for each individual molecular species and the total phosphoinositides are presented in the same way as described in Fig 2 (means and standard errors from 3 independent experiments, plotted over stimulation time in minutes). One-way ANOVA, followed by Tukey`s multiple comparison test, compared corrected normalised recoveries between different siRNA treatments for each time point. Significant result, if found, is indicated by asterisk and corresponds to p < 0.05.
(TIF)

S5 Fig Inhibitor chemical structures and IC50 values. Chemical structures of compound CRT630 (A) and CRT349 (B). IC50 values (C) were calculated in μM using recombinant proteins with *in vitro* assays.
(TIF)

S6 Fig PIP5K chemical inhibition by CRT349 in EGF-stimulated MCF10A cells. Mass spectrometry measurement of PIP_3_, PIP_2_, PIP, and PI molecular species after 30-minute incubation of MCF10A cells with varying concentrations of the PIP5K inhibitor CRT349 or its vehicle (DMSO), followed by stimulation with EGF for 1 minute. Mass spectrometry recoveries are presented as explained in Fig 6. The graphs show means and standard errors from 6 independent experiments.
(TIF)

S7 Fig PIP5K chemical inhibition by CRT349 in the absence of EGF. Mass spectrometry measurement of PIP_2_, PIP, and PI molecular species after 30-minute incubation of MCF10A cells with varying concentrations of the PIP5K inhibitor CRT349 or its vehicle (DMSO), followed by stimulation with EGF vehicle (mock stimulation) for 1 minute. Mass spectrometry recoveries are presented as explained in Fig 6. The graphs show means and standard errors from 6 independent experiments. No reliable PIP_3_ quantification was possible in the absence of EGF-stimulation.
(TIF)

S8 Fig Effect of PI3Kα-selective inhibitor A66 on EGF-dependent PIP3, p-AKT, p-EGFR, and p-MAPK response. A: MCF10A cells were treated with 2 μM A66 or its vehicle (DMSO) for 20 minutes before stimulation with EGF for 1 minute, and the molecular PIP_3_ species (C36:2, C36:1, C38:4, C38:3, C38:2), including their sum, were quantified by mass spectrometry. Recoveries were corrected over relevant ISD and for cell input over total PI / ISD PI. The graphs present means from two independent experiments, and the error bars show their range. B-D: A portion of the same samples, described in A, was used for p308/p473-AKT, p-EGFR/total EGFR, and p-MAPK immunoblotting. Fluorescent signals were normalized over loading control (βCOP). The graphs in B and C show means from 2 independent experiments and the error bars show their range. The graph in D presents means and standard errors from 3 replicate samples in 1 experiment.
(TIF)

S9 Fig PIP5K chemical inhibition by CRT630 in EGF-stimulated MCF10A cells. Mass spectrometry measurement of PIP_3_, PIP_2_, PIP, and PI molecular species after 30-minute incubation of MCF10A cells with varying concentrations of the PIP5K inhibitor CRT630 or its vehicle (DMSO), followed by stimulation with EGF for 1 minute. Mass spectrometry recoveries are presented as explained in Fig 6. The graphs show means and standard errors from 3 independent experiments.
(TIF)

S10 Fig PIP5K chemical inhibition by CRT630 in the absence of EGF. Mass spectrometry measurement of PIP_2_, PIP, and PI molecular species after 30-minute incubation of MCF10A cells with varying concentrations of the PIP5K inhibitor CRT630 or its vehicle (DMSO), followed by stimulation with EGF vehicle (mock stimulation) for 1 minute. Mass spectrometry recoveries are presented as explained in Fig 6. The graphs show means and standard errors from 3 independent experiments. No reliable PIP_3_ quantification was possible in the absence of EGF-stimulation.
(TIF)

S11 Fig PIP5K chemical inhibition by CRT349 in EGF-stimulated wild-type and PIP5K knockout cell lines. Mass spectrometry measurement of PIP_3_, PIP_2_, PIP, and PI molecular species after 30-minute incubation of parental (wild-type, wt), PIP5Kα or PIP5Kγ knockout (PIP5Kα k/o, PIP5Kγ k/o) MCF10A cells with varying concentrations of the PIP5K inhibitor CRT349 or its vehicle (DMSO), followed by stimulation with EGF for 1 minute. Mass spectrometry recoveries are presented as explained in Fig 9. The graphs show means and standard errors from 3 independent experiments. P values, if displayed, show significant result, found by F test.
(TIF)

S12 Fig PIP5K chemical inhibition by CRT349 in wild-type and PIP5K knockout cell lines in the absence of EGF. Mass spectrometry measurement of PIP_2_, PIP, and PI molecular species after 30-minute incubation of parental (wild-type, wt), PIP5Kα or PIP5Kγ knockout (PIP5Kα k/o, PIP5Kγ k/o) MCF10A cells with varying concentrations of the PIP5K inhibitor CRT349 or its vehicle (DMSO), followed by stimulation with EGF vehicle for 1 minute (mock stimulation). Mass spectrometry recoveries are presented as explained in Fig 9. The graphs show means and standard errors from 3 independent experiments. No reliable PIP_3_ signal was recovered in the absence of EGF. P values, if displayed, show significant result, found by F test.
(TIF)

S1 File Original uncropped images of immunoblots presented in Fig 1 and Fig 4. (PDF)
