# Supplementary material for: PIP5K isoforms and phosphoinositide molecular species in EGF-stimulated PIP3 synthesis: A multi-method approach using enzyme genetic suppression and chemical inhibition in MCF10A cells
Source: PLoS One. 2026 Nov 5;21(11):e0362260. doi: 10.1371/journal.pone.0362260 (PMC13739171; doi:10.1371/journal.pone.0362260)
Supplement: Supplementary file 1 [file pone.0362260.s013.pdf]

Image ID 0002248_02
05 March 2014

Uncropped and original image for Fig 1A top left panel

Image Display Parameters

Channel	Color	Minimum	Maximum	K
700	Red	533	16000	0
800	Green	392	3790	0

170 kDa
130 kDa
100 kDa
70 kDa
55 kDa
40 kDa
35 kDa

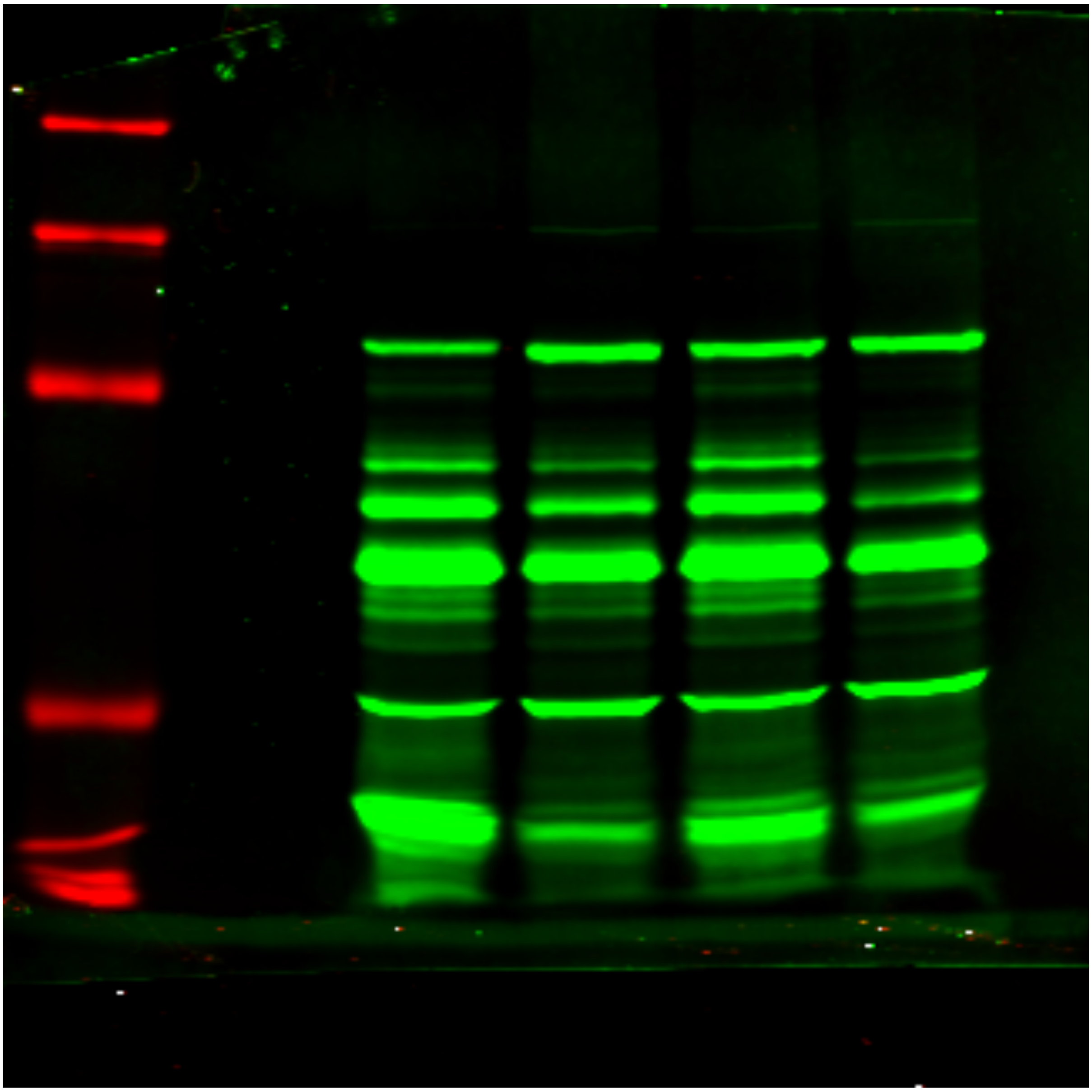

	X	X				
Lanes:	Ladder	Empty	1	2	3	4
siRNA:			Control	PIP5Kα-directed	PIP5Kγ-directed	PIP5Kα- & γ-directed

x indicates cropped lanes

Image ID 0001267_02
10 September 2013

Uncropped and original image for Fig 1A top middle panel

Image Display Parameters

Channel	Color	Minimum	Maximum	K
700	Red	2070	9270	0
800	Green	369	46100	0

170 kDa
130 kDa
100 kDa
70 kDa
55 kDa

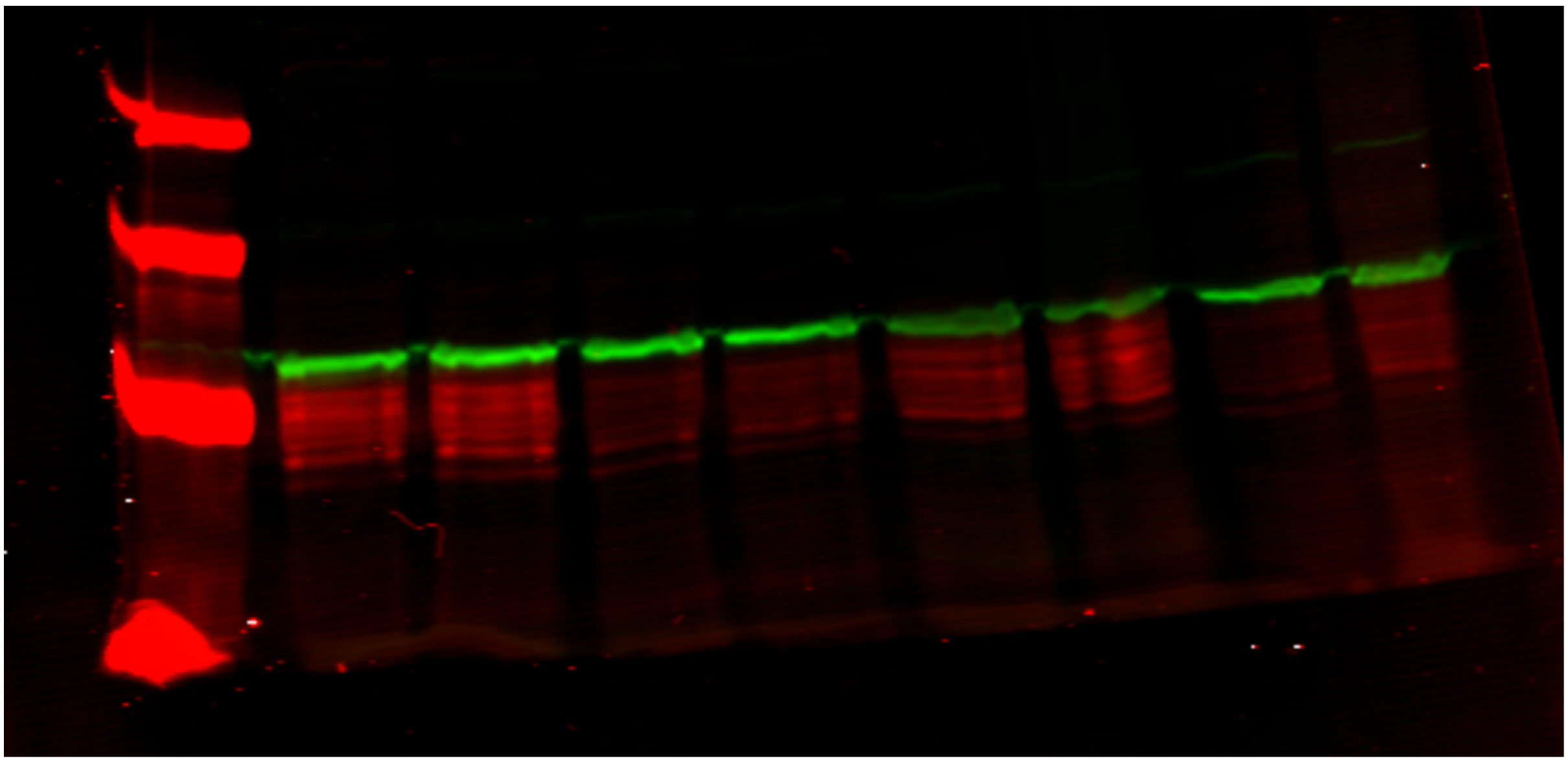

	X					X	X	X	X
Lanes:	Ladder	1	2	3	4	5	6	7	8
siRNA:		Control	PIP5Kα-directed	PIP5Kγ-directed	PIP5Kα-&γ-directed	Control	PIP5Kα-directed	PIP5Kγ-directed	PIP5Kα-&γ-directed

x indicates cropped lanes

Uncropped and original image for Fig 1A top right panel

Sample and treatment:

Lanes 1 and 6: Positive control for PIP5Kβ (K562 lysate), no siRNA (+C)

Lane 2: MCF10A cells, PIP5Kα and PIP5Kγ siRNA (α+γ)
Lane 3: MCF10A cells, PIP5Kγ siRNA (γ)
Lane 4: MCF10A cells, PIP5Kα siRNA (α)
Lane 5: MCF10A cells, non-targeting (control) siRNA (C)

Lanes 1 2 3 4 5 6

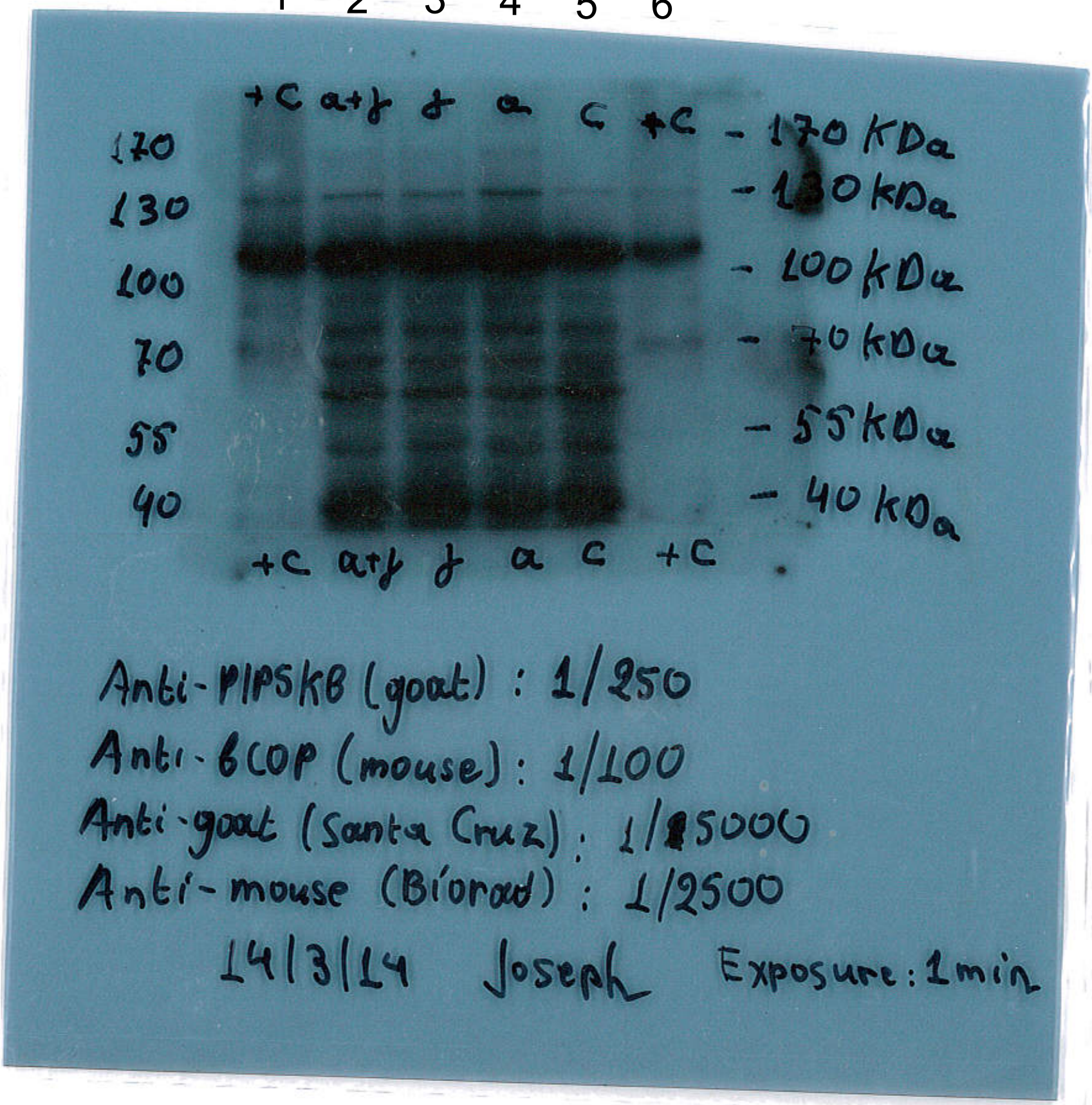

170 kDa
130 kDa
100 kDa
70 kDa
55 kDa
40 kDa

Notes on the blot refer to primary antibody dilutions (goat anti-PIP5Kβ at 1/250 and mouse anti-βCOP at 1/100), secondary antibody dilutions and manufacturer (anti-goat from Santa Cruz at 1/5000, and anti-mouse from Biorad at 1/2500)

The ladder bands at 170, 130, 100, 70, 55, and 40 kilo-daltons and the film exposure (1 minute) are also indicated

Finally, the date the blot was prepared and analyst`s name have been recorded

LI-COR®
Experience Excellence

Image ID 0003346_03
22 July 2016

Uncropped and original image for Fig 4A left panel

Image Display Parameters

Channel	Color	Minimum	Maximum	K
700	Red	323	3210	0
800	Green	707	14100	0

170 kDa
130 kDa
100 kDa
70 kDa
55 kDa
40 kDa
35 kDa

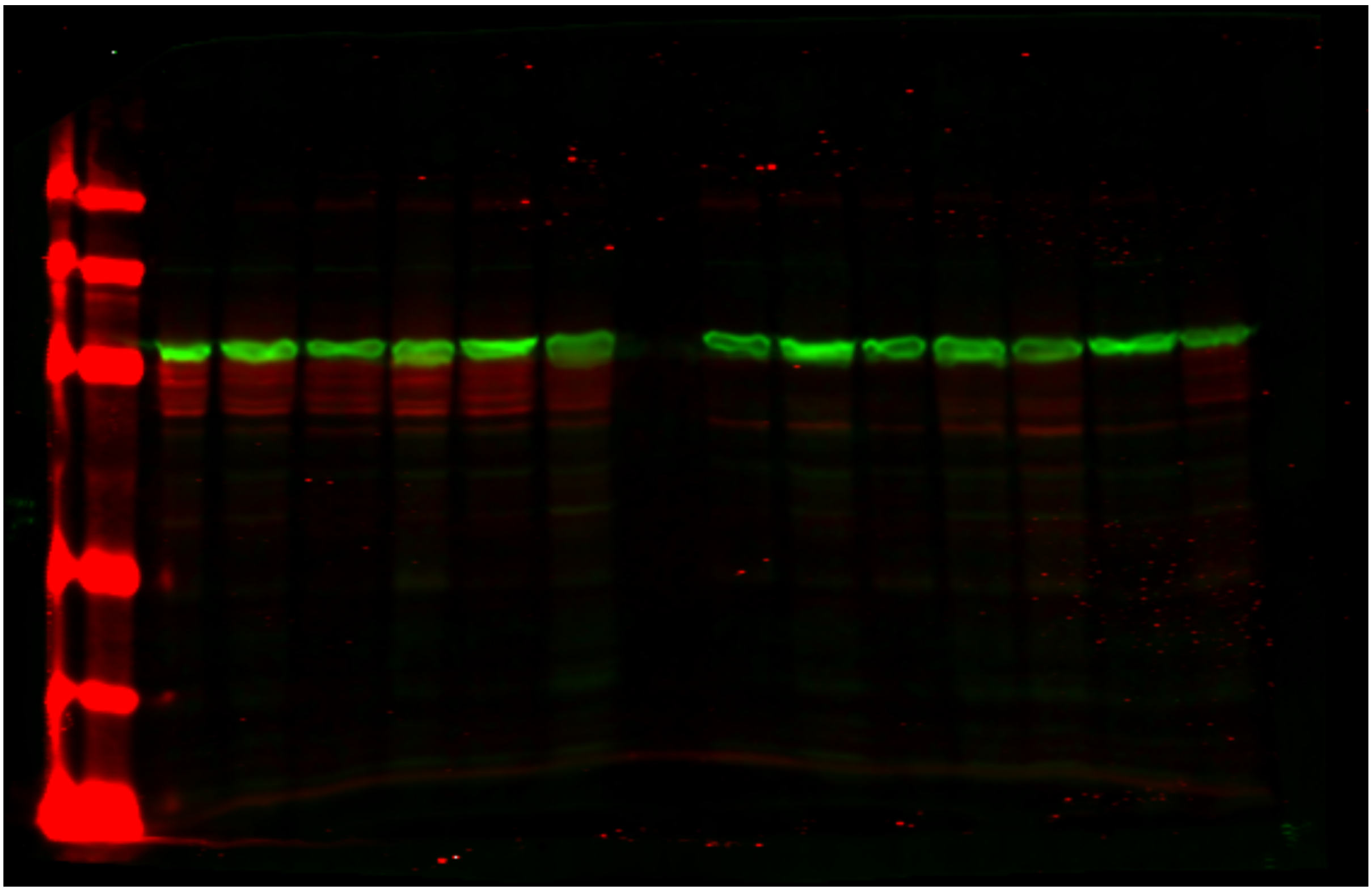

x x x x x x x x x x x x x

Lanes: Ladder 1 2 3 4 5 6 7 8 9 10 11 12 13 14

Samples (either wild-type MCF10A cells or monoclonal populations after PIP5Kγ gene editing)

Lanes 1 and 2: wild-type MCF10A cells
Lane 3: monoclonal population #2
Lane 4: monoclonal population #11
Lane 5: monoclonal population #12
Lane 6: monoclonal population #13
Lane 7: no sample (empty)
Lane 8: monoclonal population #3
Lane 9: monoclonal population #9
Lane 10: monoclonal population #19
Lane 11: monoclonal population #22
Lane 12: monoclonal population #24
Lane 13: monoclonal population #9 (repeat)
Lane 14: monoclonal population #26

From all the above monoclonal populations, #3 (lane 8) was used as the PIP5Kγ knockout MCF10A cell line in all assays.

x indicates cropped lanes

Image ID 0003347_03
21 July 2016

Image Display Parameters

Channel	Color	Minimum	Maximum	K
700	Red	314	29600	0
800	Green	953	33200	0

Uncropped and original image for Fig 4A right panel

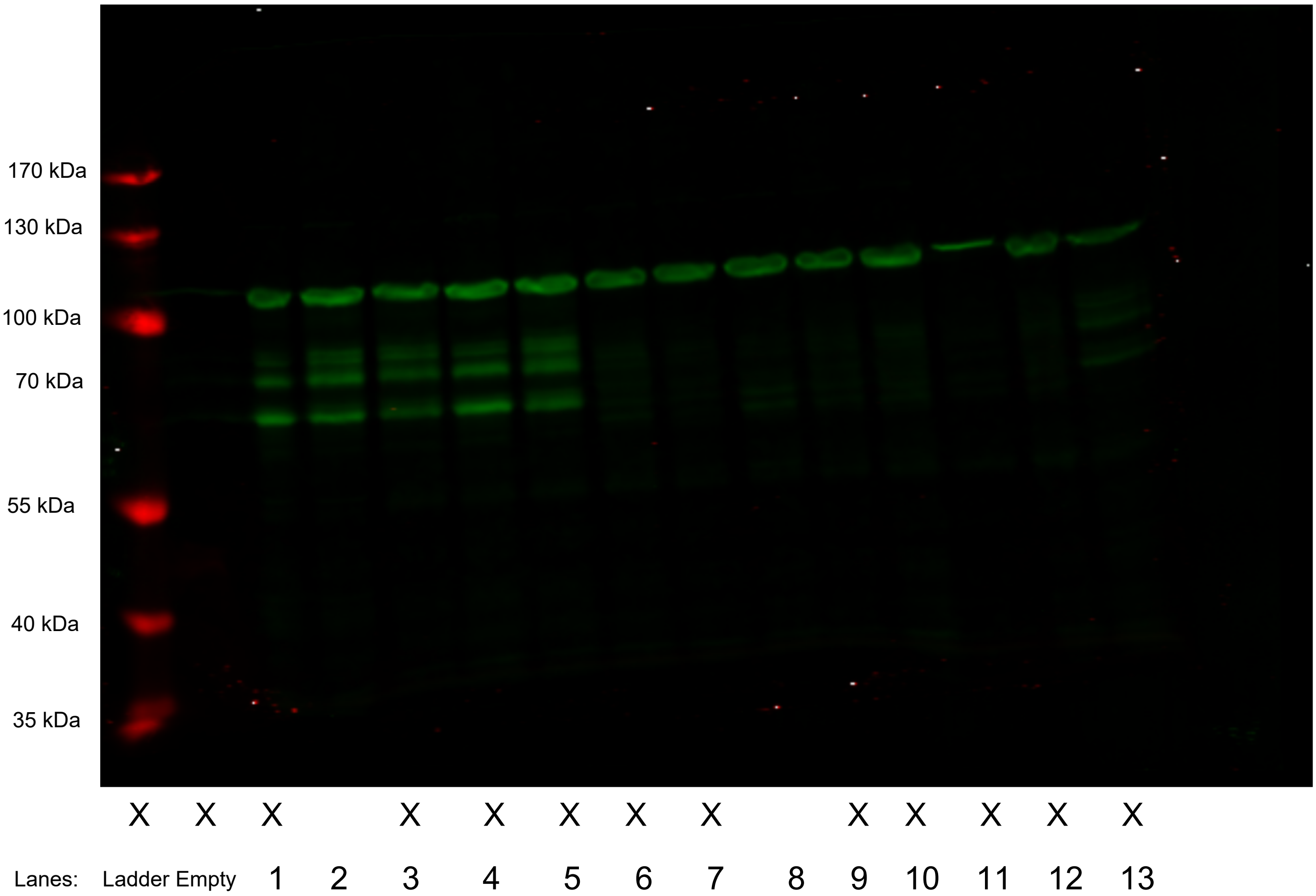

Samples (wild-type MCF10A cells or monoclonal populations after PIP5Kα knockout)

Lanes 1 and 2: wild-type MCF10A cells
Lane 3: monoclonal population #1
Lane 4: monoclonal population #4
Lane 5: monoclonal population #11
Lane 6: monoclonal population #6
Lane 7: monoclonal population #5
Lane 8: monoclonal population #10
Lane 9: monoclonal population #14
Lane 10: monoclonal population #21
Lane 11: monoclonal population #24
Lane 12: monoclonal population #25
Lane 13: monoclonal population #26

From the above monoclonal populations, #10 (lane 8) was used as the PIP5Kα knockout MCF10A cell line in all assays

x indicates cropped lanes

Uncropped and original image for Fig 4C, PIP5Kβ panel

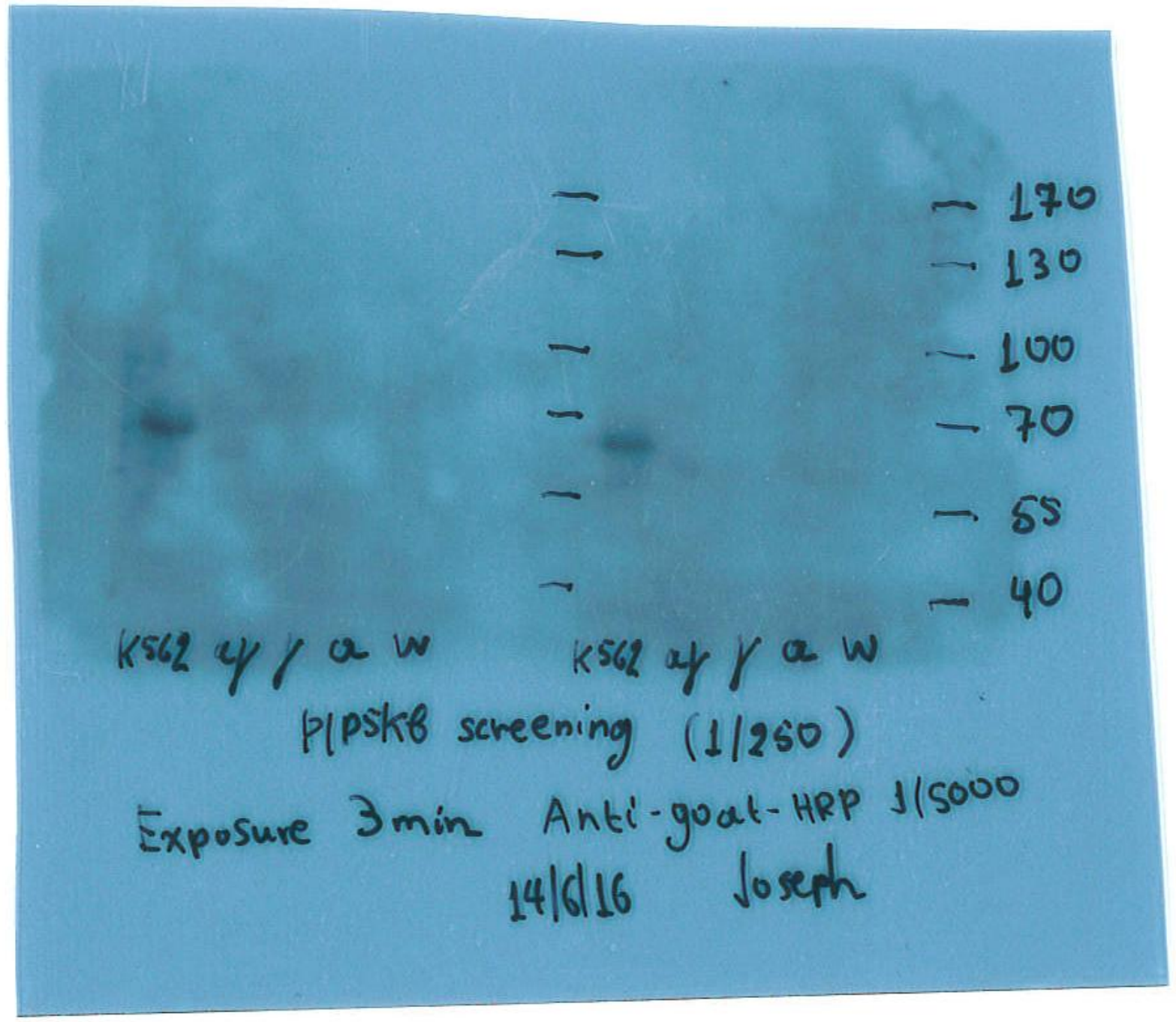

170 kDa
130 kDa
100 kDa
70 kDa
55 kDa
40 kDa

	x	x	x	x	x	x		x				x
Lanes:	1	2	3	4	5	Ladder	6	7	8	9	10	Ladder

Cell lysates from K562 cells (K562), wild-type MCF10A cells (w), or PIP5Kα- (α) or PIP5Kγ- (γ) knockout cells were screened in duplicate gels for PIP5Kβ (goat anti-PIP5Kβ at dilution 1/250, anti-goat secondary HRP-conjugated antibody at dilution 1/5000). Sample lanes, labelled as "αγ", correspond to an attempt to generate double knockout cell lines, which were not fully validated and are not presented in the manuscript. Exposure time for PIP5Kβ is indicated (3 minutes), together with date and analyst`s name. The numbers on the right (170, 130, 100, 70, 55, 40) show molecular weight in kilo-Daltons.

Lanes 1 and 6: K562 cell lysates (positive control)
Lanes 2 and 7: attempted double knockout cell lysates (not fully validated, not included in the manuscript)
Lanes 3 and 8: or PIP5Kγ- (γ) knockout cell lysates
Lanes 4 and 9: PIP5Kα- (α) knockout cell lysates
Lanes 5 and 10: wild-type MCF10A cell (w) lysates

x indicates cropped lanes

Uncropped and original image for Fig 4C, βCOP panel

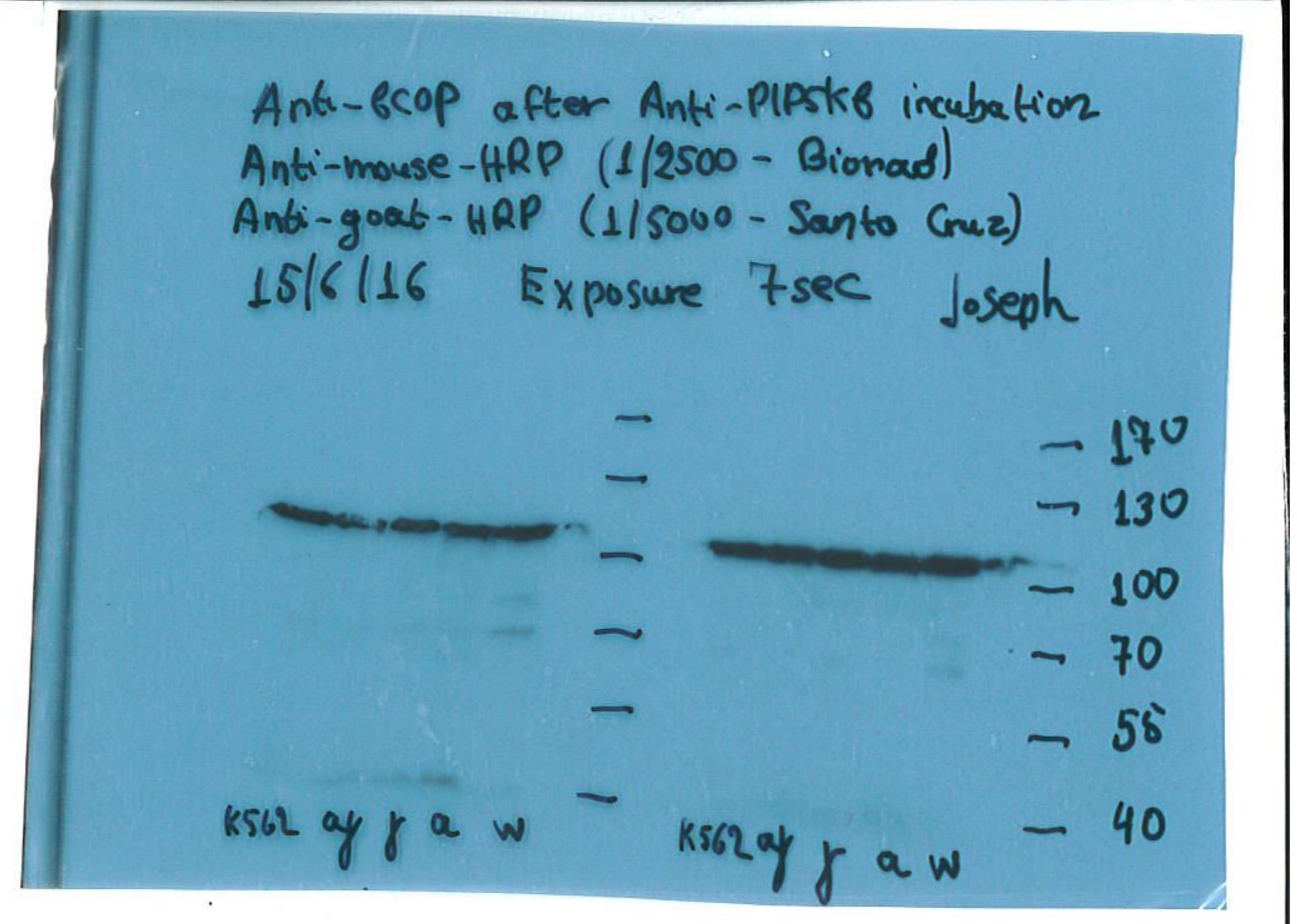

170 kDa
130 kDa
100 kDa
70 kDa
55 kDa
40 kDa

x x x x x x x x

Lanes: 1 2 3 4 5 Ladder 6 7 8 9 10 Ladder

The same blots, described above, were probed for βCOP (mouse anti-βCOP at dilution 1/100, anti-mouse secondary HRP-conjugated at dilution 1/2500). Exposure time for βCOP are indicated (7 seconds), together with date and analyst`s name. The numbers on the right (170, 130, 100, 70, 55, 40) show molecular weight in kilo-Daltons.

Lanes 1 and 6: K562 cell lysates (positive control)
Lanes 2 and 7: attempted double knockout cell lysates (not fully validated, not included in the manuscript)
Lanes 3 and 8: or PIP5Kγ- (γ) knockout cell lysates
Lanes 4 and 9: PIP5Kα- (α) knockout cell lysates
Lanes 5 and 10: wild-type MCF10A cell (w) lysates

x indicates cropped lanes
